# Phosphorylation of presynaptic PLPPR3 controls synaptic vesicle release

**DOI:** 10.1016/j.isci.2025.113435

**Published:** 2025-08-27

**Authors:** Cristina Kroon, Shannon Bareesel, Gerard Aguilar Perez, Domonkos Nagy-Herczeg, Dimitra Ranti, Vasiliki Syropoulou, Sandra Coveney, Marieluise Kirchner, Niclas Gimber, Willem Bintig, Annika Brosig, Georg Braune, Kathrin Textoris-Taube, Timothy A. Zolnik, Philipp Mertins, Jan Schmoranzer, Dragomir Milovanovic, George Leondaritis, Britta J. Eickholt

**Affiliations:** 1 Institute of Molecular Biology and Biochemistry, Charité-Universitätsmedizin Berlin, Charitéplatz 1, 10117 Berlin, Germany; 2 Laboratory of Molecular Neuroscience, German Center for Neurodegenerative Diseases (DZNE), 10117 Berlin, Germany; 3 Cell Signaling Technology, 3 Trask Lane, Danvers, MA 01923, USA; 4 Core Unit Proteomics, Berlin Institute of Health at Charité-Universitätsmedizin Berlin and Max Delbrück Center for Molecular Medicine, Berlin, Germany; 5 Advanced Medical Bioimaging Core Facility, Charité-Universitätsmedizin Berlin, Charitéplatz 1, 10117 Berlin, Germany; 6 Core Facility High Throughput Mass Spectrometry, Charité-Universitätsmedizin Berlin, Charitéplatz 1, 10117 Berlin, Germany; 7 Department of Pharmacology, Faculty of Medicine, School of Health Sciences, University of Ioannina, 45110 Ioannina, Greece; 8 Institute of Biosciences, University Research Center Ioannina, University of Ioannina, Ioannina, Greece

**Keywords:** biochemistry, neuroscience, cell biology

## Abstract

Phospholipid-phosphatase-related protein 3 (PLPPR3) belongs to a family of transmembrane proteins highly expressed in the nervous system where it regulates critical axonal growth processes during guidance, filopodia formation, and branching. However, little is known regarding its role in synapses and the signaling events regulating PLPPR3 function. Here, we identify 26 high-confidence phosphorylation sites in the intracellular domain of PLPPR3 using mass spectrometry. Biochemical characterization established one of these—S351—as a *bona fide* phosphorylation site of protein kinase A (PKA). PLPPR3 is enriched at presynaptic terminals, and deletion of PLPPR3 results in increased depolarization-induced synaptic vesicle release in hippocampal neurons. This tonic inhibitory signal toward depolarization-induced presynaptic activity is corrected by expression of PLPPR3 intracellular domain, but not a S351A phospho-dead mutant, in *Plppr3*^−/−^ hippocampal neurons. We propose that PLPPR3 phosphorylation under the control of PKA activity is a signaling integrator of presynaptic activity in hippocampal neurons.

## Highlights

• Discovery of 26 phosphorylation sites in C terminus of the transmembrane protein PLPPR3
• PKA mediates phosphorylation of PLPPR3 at Ser351
• Ser351 phosphorylation mediates the interaction with the synaptic protein BASP1
• Ser351 phosphorylation regulates KCl-induced synaptic vesicle release

## Graphical abstract

**Figure:**
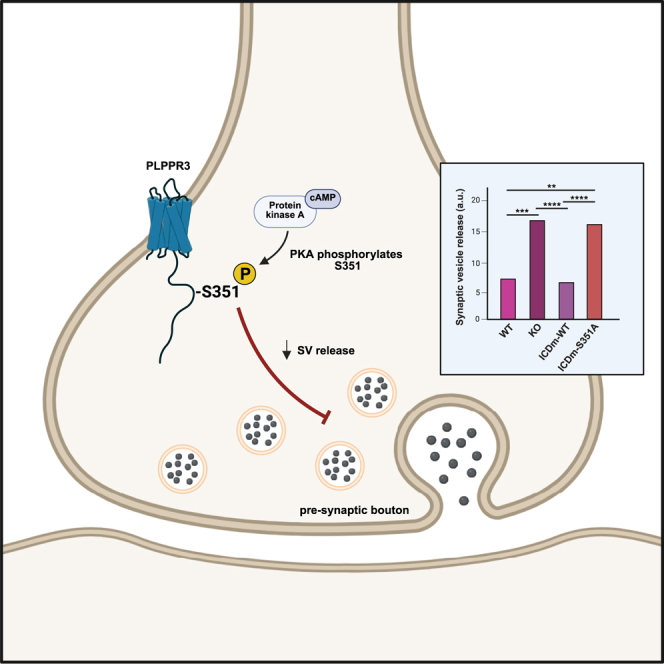

## Introduction

Phospholipid-phosphatase-related protein 3 (PLPPR3), previously also known as plasticity-related gene 2, belongs to a family of membrane proteins (PLPPR1–5) with described functions in neuronal growth and synaptic transmission.^1^ All PLPPRs share highly conserved six transmembrane domains and much less conserved intracellular domains (ICDs) of various lengths. This topology makes them ideally suited to integrate cell-extrinsic and cell-intrinsic signals involved in orchestrating growth and plasticity.

PLPPR3 is highly expressed during early stages of neuronal development, where it controls guidance of thalamic axons and fine-tunes axonal branching by inducing filopodia.^2^^,^^3^^,^^4^ In developing glutamatergic and GABAergic neurons, it localizes to the axonal plasma membrane, where it forms clusters, and a previous work in our lab has demonstrated that filopodia form at or near these PLPPR3 clusters.^3^ Our recent data further indicate that some PLPPR3 expression persists in adults, where it is highest in the striatum and nucleus accumbens.^5^ Moreover, PLPPR3 is found in synaptosomal fractions isolated from various adult brain areas.^5^ Thus, it is likely that PLPPR3 has hitherto unknown synaptic functions that extend beyond strictly growth-related developmental phases.^5^

The functions of PLPPR3 are mediated by its long ICD, which is ideally suited to form signaling complexes. Indeed, PLPPR3-based filopodia formation requires binding and inhibition of phosphatase and tensin homolog (PTEN) via its ICD, and PLPPR3-dependent thalamocortical targeting of growth cones relies on binding to radixin via its ICD.^2^^,^^3^ How these interactions are regulated is currently not known.

Protein phosphorylation is an important regulatory mechanism that essentially controls most cellular processes. It is a rapid and reversible way of regulating proteins post-translationally. The addition of a phosphoryl group, catalyzed by kinases, adds negative charges to the protein and leads to changes in its binding properties or conformation, thereby inducing a variety of outcomes such as changes in protein localization.^6^^,^^7^^,^^8^ Due to its rapid and pervasive way of changing protein structure and function, phosphorylation is central to intracellular signaling pathways. Curiously, prediction tools suggest that a large number of residues in the PLPPR3 ICD may be phosphorylated (NetPhos3.1; https://services.healthtech.dtu.dk/services/NetPhos-3.1/), indicating the possibility for PLPPR3 to act as a signaling hub. To date, phosphorylation of PLPPRs has not been studied.

Here, we identify 26 high-confidence phosphorylation sites in the ICD of PLPPR3 using mass spectrometry. One of the identified PLPPR3 sites is phosphorylated by protein kinase A (PKA) at serine 351, and this phosphorylation event regulates its binding to brain acid soluble protein 1 (BASP1, also known as CAP23 or NAP22), a growth- and synapse-associated signaling molecule.^9^ PLPPR3 localizes to presynaptic boutons along axons, and its phosphorylation at S351 regulates synaptic vesicle release upon KCl-induced depolarization in primary neurons. We propose PLPPR3 as a signaling integrator at presynapses in the central nervous system (CNS).

## Results

### The ICD of PLPPR3 is highly phosphorylated

**Figure 1:**
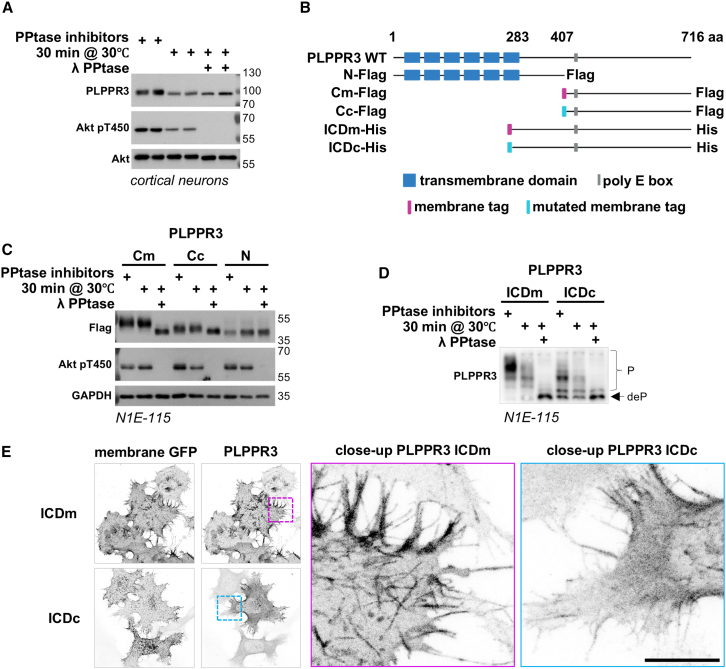
PLPPR3 intracellular domain is phosphorylated (A) Phosphorylation of PLPPR3 was assessed in DIV9 primary cortical neurons by band shift on SDS-PAGE following dephosphorylation with lambda phosphatase (PPtase; 30 min at 30°C). (B) Schematic of PLPPR3 WT and PLPPR3 variants used in this study. (C) Phosphorylation of truncated PLPPR3 variants was assessed by band shift on SDS-PAGE/western blot. (D) Phosphorylation of PLPPR3 ICD variants expressed in N1E-115 cells was assessed by PhosTag SDS-PAGE. (E) Localization of PLPPR3 ICD variants in NIE-115 cells. Membrane-tagged GFP was used to visualize membranes. Scale bars, 10 μm. ICD, intracellular domain.

We tested phosphorylation of PLPPR3 in primary cortical neurons at days *in vitro* (DIV) 9 when its expression peaks.^3^ Following lambda phosphatase treatment to non-selectively remove all phosphoryl-groups, we identified a small but noticeable band shift in the migration of PLPPR3 when compared to the untreated samples (Figure 1A). We next used the phosphorylation prediction tool NetPhos3.1 (https://services.healthtech.dtu.dk/services/NetPhos-3.1/) to investigate which residues of PLPPR3 could be targeted by phosphorylation. Transmembrane domains and extracellular loops were not considered in our analyses due to their inaccessibility to kinases. Using a stringent probability of >0.75, a total of 43 residues were predicted to be phosphorylated, with 39 of them located in the PLPPR3 ICD and four located in the intracellular loops (data not shown). To directly test the accuracy of the predictions, we generated multiple PLPPR3 variants (Figure 1B). All intracellular fragments were modified with a myristoylation/palmitoylation tag for membrane localization (Cm or ICDm), or a mutated tag for cytosolic localization (Cc or ICDc), since proximity to the plasma membrane may play an important role in phospho-regulation.^10^ We expressed the PLPPR3 variants in N1E-115 neuroblastoma cells and analyzed their phosphorylation status by western blotting (Figure 1C). Interestingly, the membrane-tagged PLPPR3-Cm variant exhibited a bigger band shift in response to phosphatase treatment when compared to the PLPPR3-Cc variant (Figure 1C).

Next, we utilized ICD variants along with PhosTag SDS-PAGE analysis. PhosTag is a phosphate-binding molecule that traps phosphorylated groups in the gel, allowing separation of proteins based on their phosphorylation state.^11^ Using lambda phosphatase treatment, we found that the membrane-tagged ICDm exists in an almost entirely phosphorylated state in cells, indicated by multiple strong phospho-bands and a very weak non-phospho band (Figure 1D, left three lanes). In contrast, the primarily cytosolic ICDc displayed fewer higher-order phosphorylation bands and an evident non-phospho band (Figure 1D, right three lanes). Immunolabeling in N1E-115 cells confirmed the localization of these variants to the plasma membrane (ICDm) or cytosol (ICDc, Figure 1E). These results indicate multiple phosphorylation events in the ICD of PLPPR3 and suggest that proximity to the plasma membrane is crucial for the dynamic regulation of its phosphorylation status.

**Figure 2:**
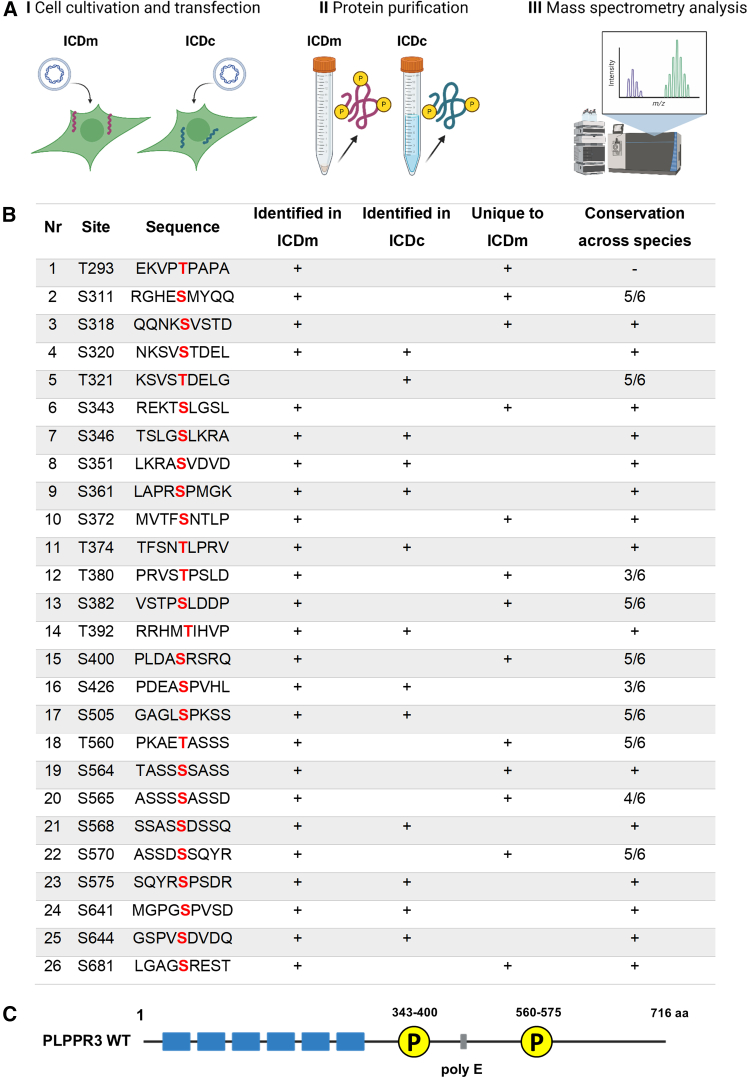
PLPPR3 phosphosite characterization (A) Experimental workflow. (B) List of phosphorylated residues in ICDm and ICDc and their conservation across species. Phosphorylated residues are marked in red. Conservation of the phosphorylated residue was analyzed by sequence alignment across six species: mouse (*Mus musculus*), human (*Homo sapiens*), zebrafish (*Danio rerio*), tropical clawed dog (*Xenopus tropicalis*), rhesus monkey (*Macaca mulatta*), and chicken (*Gallus gallus*). Conserved phosphorylation sites are marked with + and non-conserved with −; the exact ratio of conserved/total is given. The conservation analysis was performed with COBALT tool (COBALT:Multiple Alignment Tool [nih.gov]). (C) Schematic of PLPPR3 with phosphorylation hotspots.

To characterize phosphorylation sites in the ICD of PLPPR3, we purified PLPPR3 ICDm and ICDc from HEK293T lysates using His tag affinity chromatography (Figure 2A). Mass spectrometry analysis revealed 26 high-confidence phosphorylation sites within the PLPPR3 ICD (Figure 2B). Half of these phosphorylation sites were unique to the membrane-tagged PLPPR3 ICDm variant, one was specific to the cytosolic PLPPR3 ICDc variant, and twelve were present in both variants regardless of subcellular localization. The membrane-proximal intracellular region contained 15 phosphorylation sites, with a particularly dense phosphorylation cluster around residues 343–400. In the most distal part of the intracellular region, phosphorylation events were densely clustered between amino acid residues 560–575 (Figure 2C).

Altogether, our experiments demonstrate that the ICD of PLPPR3 bears distinct phosphorylation clusters flanking the acidic polyE box and that it is highly phosphorylated, with specific phosphorylation patterns influenced by proximity to the plasma membrane.

### PKA phosphorylates PLPPR3 S351 and regulates its binding to BASP1

**Figure 3:**
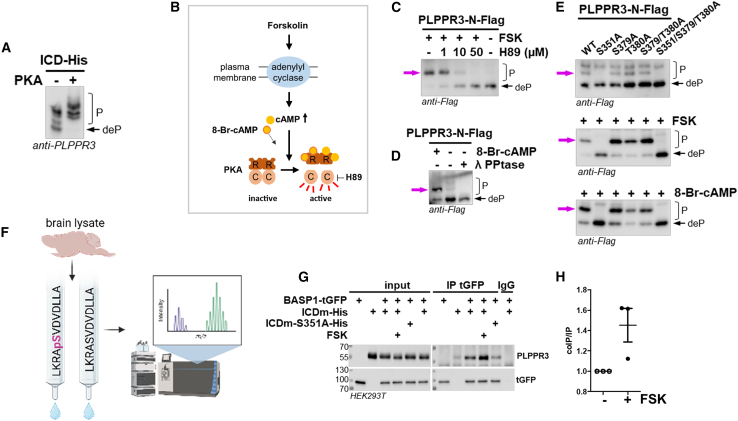
PKA phosphorylates PLPPR3 at S351, which regulates binding to BASP1 (A) *In vitro* phosphorylation of PLPPR3 ICD by PKA. Purified ICD from *E.coli* was incubated with PKA in the presence of ATP for 2 h at 30°C. Samples were analyzed by PhosTag western blot. (B) Graphic illustration of PKA activation in cells and the mechanism of action of drugs used. (C) Phosphorylation of PLPPR3-N-Flag is altered in cells by PKA-modulating drugs. N1E-115 cells were transfected with PLPPR3-N-Flag and serum-starved overnight. Cells were treated with PKA inhibitor H89 for 1 h, followed by stimulation with 30 μM Forskolin (FSK) for 5 min. Samples were analyzed by PhosTag western blot. (D) Phosphorylation of PLPPR3-N-Flag is altered in cells by a cyclic adenosine monophosphate (cAMP) analog. N1E-115 cells were transfected with PLPPR3-N-Flag and serum-starved overnight. Cells were stimulated with 1 mM 8-Bromoadenosine 3',5'-cyclic adenosine monophosphate (8-Br-cAMP) for 5 min and analyzed by PhosTag western blot. Lambda-phosphatase-treated sample shows unphosphorylated protein. (E) N1E-115 cells were transfected with indicated phospho-mutants and protein lysates were analyzed by PhosTag western blot. Phosphorylation pattern of PLPPR3-N-Flag phospho-mutants under steady-state conditions (top). Phosphorylation of PLPPR3-N-Flag phospho-dead mutants following treatment with 30 μM Forskolin (middle), or 1 mM 8-Br-cAMP (bottom), for 5 min. Magenta arrows point to the changes in phosphorylation pattern. (F) Experimental workflow. Affinity columns with immobilized pS351 and S351 peptides were incubated with brain lysate 1 h, eluted, and eluates were analyzed for interaction partners using mass spectrometry. (G) Co-immunoprecipitation of BASP1 and PLPPR3 ICDm. Protein lysates were prepared from HEK293T cells expressing BASP1-tGFP and ICDm-His, and BASP1 was immunoprecipitated using tGFP antibody. Cells were stimulated with 30 μM Forskolin for 10 min, where applicable. (H) Quantification of protein bands from (G). The amount of co-immunoprecipitated PLPPR3 ICDm is expressed over precipitated BASP1 amount. All control values were normalized to 1. Data are represented as mean ± SEM.

Next, we conducted an *in vitro* phosphorylation assay using purified PLPPR3 ICD from *Escherichia coli* and commercially available kinases, analyzing protein phosphorylation by PhosTag SDS-PAGE. This analysis revealed that PKA can phosphorylate the purified PLPPR3 ICD, as evidenced by a prominent shift toward higher-order phosphorylation bands (Figure 3A).

Based on its known kinase recognition motif,^12^ PLPPR3 ICD shows four potential PKA target sites. Our phospho-mass spectrometry analysis identified two of these, S351 and T380, as phosphorylated sites in HEK293T cells (Figure 2B). Additionally, S379 has been identified in previous proteomics studies,^13^ therefore, we considered it as well. The fourth site, S581, was not yet identified in any mass spectrometry analysis and therefore not further considered. To test whether PLPPR3 S351, T380, and/or S379 are targeted by PKA in cells, we made use of the PLPPR3-N-Flag variant (see Figure 1B) and pharmacological manipulation of PKA activity (Figure 3B).^14^ Analyses by PhosTag revealed that stimulation with Forskolin leads to phosphorylation of PLPPR3-N, as indicated by the appearance of a phospho-band, which was antagonized by presence of the PKA inhibitor H89 (Figure 3C). Furthermore, 8-Bromoadenosine 3',5'-cyclic adenosine monophosphate (8-Br-cAMP) induces the phosphorylation of PLPPR3-N (Figure 3D), indicating that PKA can phosphorylate PLPPR3 in a cAMP-dependent manner. As in previous experiments, we used lambda phosphatase treatment as reference for the non-phosphorylated form of PLPPR3-N variant.

We used site-directed mutagenesis to generate non-phosphorylatable (S→A) mutants of the three potential PKA motifs, as well as a double and a triple mutant, to identify the relevant PKA site(s). We observed that the S351A as well as the S351A/S379A/T380A triple mutant lacked a phosphorylation band (Figure 3E). Furthermore, our analysis shows that S351A and the triple mutant cannot be phosphorylated in response to Forskolin or 8-Br-cAMP (Figure 3E). Taken together, these results demonstrate that S351 is a PKA phosphorylation site in the PLPPR3 ICD.

Phosphorylation can regulate the nature and strength of protein-protein interactions and recruitment of signaling effectors.^15^ Therefore, we established an unbiased screen and used affinity columns conjugated with PLPPR3 S351 phospho- (LKRA**pS**VDVDLLA) and non-phospho- (LKRA**S**VDVDLLA) peptides to enrich for binding partners from adult brain lysate. Bound proteins were then characterized using mass spectrometry (Figure 3F). This experimental approach identified BASP1, a signaling protein complementing the known spatiotemporal expression as well as functions of PLPPR3.^9^^,^^16^^,^^17^ Interestingly, BASP1 was enriched in the phospho-column compared to the non-phospho column, suggesting favored binding to phosphorylated S351 peptide. Using co-immunoprecipitation, we directly tested the interaction following overexpression of PLPPR3 IDCm and BASP1-turboGFP in HEK293T cells. Indeed, PLPPR3 ICDm immunoprecipitated with BASP1 under steady state conditions (Figure 3G), and this interaction significantly increased after Forskolin stimulation. In contrast, non-phospho PLPPR3-ICDm-S351A showed decreased binding to BASP1 (Figure 3G). These experiments establish BASP1 as an interaction partner of PLPPR3 and indicate that the PLPPR3-BASP1 interaction is regulated by PKA-induced PLPPR3 phosphorylation at S351.

BASP1 is a growth-associated protein described to regulate axon development and regeneration, as well as synaptic function.^16^^,^^18^ In HEK293T cells, PLPPR3 ICDm and BASP1 are co-localized in the cell peripheral structures and occasionally enriched at the tips of filopodia (Figure S1A). We thus reasoned that PLPPR3 phosphorylation at S351 and BASP1 interaction may relate to the known filopodia induction function of PLPPR3.^1^^,^^3^^,^^4^ To test this idea, we expressed WT PLPPR3, which induces filopodia formation in cells,^19^ and compared this activity to phospho-dead (S351A) and phospho-mimic (S351D) variants. All PLPPR3 phospho-mutants exhibited equivalent plasma membrane localization as the WT protein, which is necessary for PLPPR-induced filopodia formation^1^ (Figure S1B). However, there were no differences in filopodia density between the phospho-mutants and the WT (Figure S1C). We conclude that phosphorylation of PLPPR3 at S351 does not regulate filopodia formation.

### Endogenous PLPPR3 is phosphorylated at S351 in neurons and adult brain tissue

Although PLPPR3 S351 does not control filopodia formation, conservation analysis suggests that it may be a functionally relevant site. Therefore, we decided to raise an anti-PLPPR3 pS351 phospho-site-specific antibody to facilitate further functional analyses. Following affinity purification, we rigorously tested the specificity of this antibody toward the PLPPR3 pS351 epitope using a phospho-dead mutant (PLPPR3 S351A), lambda phosphatase treatments, as well as *WT* and *Plppr3*^−/−^ neuron lysates. Together, these findings indicate that anti-pS351-PLPPR3 specifically recognizes the phosphorylated serine 351 of PLPPR3 in western blot applications (Figures S2A and S2B).

**Figure 4:**
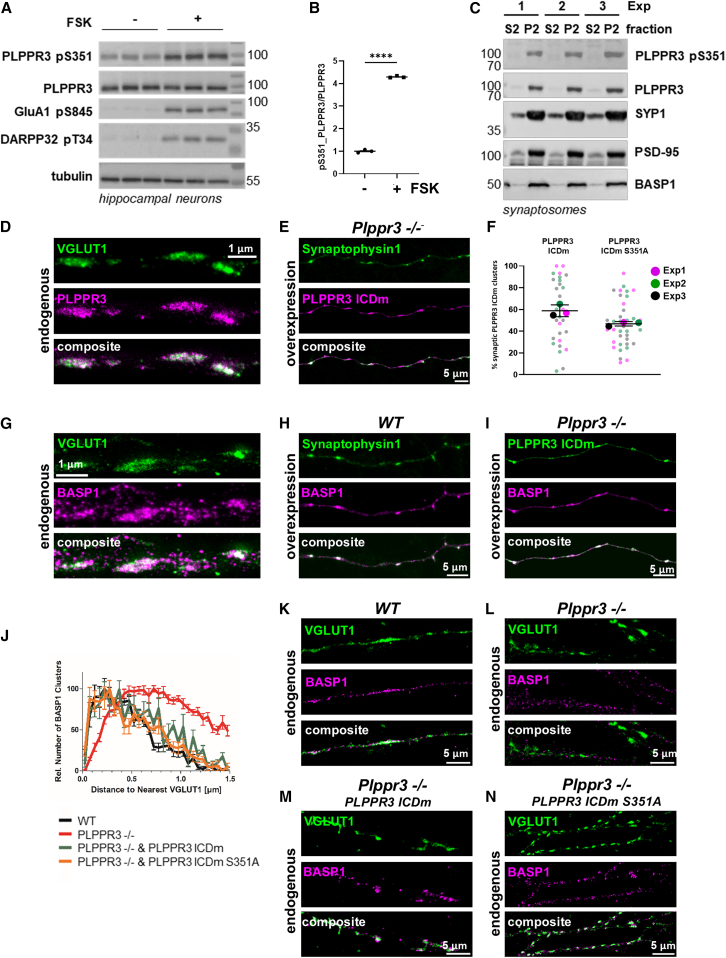
PLPPR3 and BASP1 localize to the presynaptic terminal (A) Phosphorylation of PLPPR3 S351 can be triggered in neurons. DIV8 primary hippocampal neurons were stimulated with Forskolin (30 μM, 5 min), and analyzed by western blot using indicated antibodies. (B) Quantification of the results in A (*n* = 3). Data are represented as mean ± SEM. ∗∗∗∗*p* < 0.0001, unpaired t test. (C) Localization of PLPPR3 and pS351 to synaptosomal fractions. Crude synaptosomes were prepared from adult mouse brain and analyzed by western blot. S2, cytosolic fraction; P2, crude synaptosomes; SYP1, Synaptophysin1. (D) Localization of PLPPR3 to presynaptic terminals in DIV16 *WT* primary hippocampal neurons analyzed by STED microscopy. PLPPR3 was stained with our custom made anti-PLPPR3 antibody in combination with anti-VGLUT1. (E) Co-localization of Synaptophysin1 and PLPPR3 ICDm following overexpression in primary hippocampal neurons. Neurons were transfected with recombinant proteins at DIV1 and analyzed at DIV7. (F) Quantification of PLPPR3 ICDm clusters inside Synaptophysin1-positive synapses. Synaptophysin1 was co-expressed with PLPPR3 ICDm or PLPPR3 ICDm S351A. Graph shows PLPPR3 ICDm (WT or S351A) in Synaptophysin1-positive synapses. *N* = 3, *n* ≥ 7 neurons. Data are represented as mean ± SEM. (G) Localization of BASP1 to presynaptic terminals in DIV16 *WT* primary hippocampal neurons analyzed by STED microscopy. BASP1 was stained with the custom made anti-BASP1 antibody in combination with anti-VGLUT1. (H) Co-localization of Synaptophysin1 and BASP1 in *WT* hippocampal neurons. (I) Co-localization of PLPPR3 ICDm and BASP1 in *Plppr3−/−* hippocampal neurons. In (H) and (I), neurons were transfected with recombinant proteins at DIV1 and imaged at DIV7. Scale bars, 5 μm. (J) Distance of BASP1 clusters to nearest VGLUT1 clusters (pre-synapses) in *WT* and *PLPPR3 −/−* neurons, and in *PLPPR3 −/−* neurons expression PLPPR3 ICDm or PLPPR3 ICDm S351A. Data are represented as mean ± SEM. (K–N) Examples of *WT* and *PLPPR3 −/−* neurons, and *PLPPR3 −/−* neurons expressing PLPPR3 ICDm or PLPPR3 ICDm S351A. Neurons were stained with anti-VGLUT1 and anti-BASP1 antibodies. Scale bars, 5 μm.

To verify that this site is phosphorylated in neurons, we first analyzed its temporal pattern in primary hippocampal cultures. Total PLPPR3 protein levels are increased between 5 and 9 DIV, coinciding with the time axons undergo branching.^3^ PLPPR3 pS351 was phosphorylated at all timepoints tested, generally following total PLPPR3 levels (Figure S3A). In DIV8 hippocampal neurons, PKA activity triggered by Forskolin led to a robust increase in pS351 (Figure 4A; quantification in Figure 4B), along with increased phosphorylation of known PKA substrates, GluA1 and DARPP32.^20^^,^^21^ Notably, PLPPR3 S351 phosphorylation was triggered more robustly at later neuronal stages (i.e., DIV9) when compared to earlier neuronal stages (i.e., DIV5; Figure S3B). Recent work suggests that PLPPR3 expression ensues in the adult brain and may also have roles in adult synapses.^5^ Therefore, we tested whether PKA-dependent S351 phosphorylation can also be induced in adult brain tissue, where structural and functional integrity of intrinsic synaptic connections is maintained.^22^ Indeed, stimulation of an acute brain slice with Forskolin led to an increase in PLPPR3 pS351 levels, similar to GluA1 pS845, a known synaptic PKA target (Figure S3B).^20^

We then verified the synaptic localization of PLPPR3 using synaptosomes prepared from adult mouse brain and found that PLPPR3 pS351 was enriched in synaptosomal fractions, along with known synaptic markers (Figure 4C).^5^ Together, these experiments confirm that phosphorylation of PLPPR3 at S351 may be a functionally relevant regulatory site in the brain and points to a possible involvement in synaptic functions.

### PLPPR3 and BASP1 localize in presynaptic clusters along axons

Next, we utilized stimulated emission depletion (STED) microscopy to examine the distribution of endogenous PLPPR3 in hippocampal neurons at DIV16, a stage characterized by the presence of mature synapses. This analysis revealed a striking localization of PLPPR3 in distinct puncta along the axon, clearly co-localizing with the presynaptic marker VGLUT1 (Figure 4D). Importantly, a similar localization was observed in DIV7 *Plppr3*^−/−^ hippocampal neurons transfected with the intracellular, membrane-tagged PLPPR3 ICD variant (PLPPR3 ICDm-His). In this case, PLPPR3 ICDm fluorescent puncta were observed co-localizing with co-transfected Synaptophysin-GFP (Figure 4E). This result demonstrates that the isolated membrane-tagged PLPPR3 ICD can recapitulate the localization of endogenous PLPPR3 in presynaptic boutons. A similar localization was also observed for the S351A mutation variant of PLPPR3 ICDm (Figure 4F).

In order to establish whether PLPPR3 and BASP1 co-localize in presynaptic clusters along axons, we generated an anti-BASP1 antibody. In western blot and immunofluorescence applications, anti-BASP1 specifically recognizes a band of approximately 50 kD and exogenously expressed BASP1 in HEK293 cells, respectively (Figures S4A and S4B).^23^ Next, we utilized STED microscopy to examine the distribution of endogenous BASP1 in mature DIV16 hippocampal neurons. BASP1 was localized in distinct puncta along the axon, which co-localized with the presynaptic marker VGLUT1 (Figure 4G). Co-expression of a tagged BASP1 variant (BASP1-Flag) with Synaptophysin1-GFP revealed co-localization in clusters in axons of hippocampal neurons (Figure 4H). These results verify enrichment of BASP1 in presynaptic boutons in mature (DIV14) hippocampal neurons. In synaptosomal preparations**,** we also identified a significant enrichment of BASP1 in synaptic compartments (Figure 4C). In the final set of experiments, we demonstrate that PLPPR3 ICDm co-localized with BASP1 in distinct puncta along axons following overexpression of both proteins in *Plppr3*^−/−^ neurons (Figure 4I). Nearest Neighbor (NN) analysis assessing the spatial relationship of either BASP1 or PLPPR3 with pre- or post-synaptic markers Synaptophysin and Drebrin, respectively, confirmed the pre-synaptic localization of both proteins. In addition, NN analysis between BASP1 and PLPPR3 revealed co-clustering of both proteins, as indicated by a prominent NN peak at ∼100 nm (Figure S5). We also analyzed if PLPPR3 regulates BASP1 enrichment at presynaptic structures and performed NN analyses of endogenous BASP1 signals and VGLUT1 in *WT* and *Plppr3*^−/−^ neurons (Figures 4J–4N). This quantification revealed that in *Plppr3*^−/−^ neurons the BASP1 distance to VGLUT1 was slightly shifted from peak distances of 200–500 nm when compared to *WT* neurons. Interestingly, expression of either PLPPR3 ICD or PLPPR3 ICDm S351A in *Plppr3*^−/−^ neurons reconstitute normal BASP1 localization to *WT* neurons (Figures 4J, 4M, and 4N). These data suggest that PLPPR3, regardless of PKA phosphorylation at S351, is necessary for recruitment or retention of BASP1 in presynaptic VGLUT1 clusters at basal conditions.

### PLPPR3 phosphorylation at S351 controls synaptic vesicle release

The striking shift of PLPPR3 localization during development from diffuse or punctate labeling of axonal shaft and axonal filopodia toward VGLUT1- and Synaptophysin-labeled axonal boutons in synapses, together with its localization to adult brain synaptosomes. are consistent with its possible function in synaptic transmission. Since an important aspect of synaptic communication relates to the fusion of synaptic vesicles (SVs) with the presynaptic plasma membrane and subsequent neurotransmitter release,^24^ we asked if PLPPR3 controls synaptic transmission by affecting the release of neurotransmitters. We thus used a Synaptophysin-pHluorin (Syph-pHluorin) probe, consisting of a pH-sensitive fluorophore tagged to the luminal side of Synaptophysin, to analyze the SV release (Figure S6A).^25^

**Figure 5:**
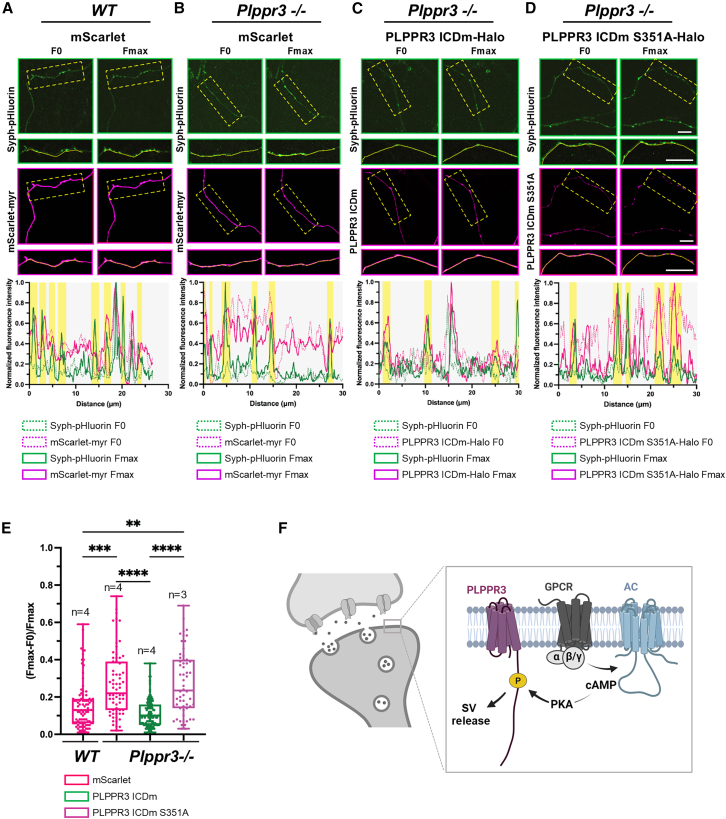
PLPPR3 phosphorylation at S351 controls synaptic vesicle release (A–D) Exemplary images of a pHluorin assay performed on *WT* or *Plppr3−/−* neurons (DIV 14) transfected with Syph-pHluorin and mScarlet-myr (A, B), PLPPR3 ICDm-Halo (C), or with PLPPR3 ICDm S351A-Halo (D). Before stimulation (F0, left), Syph-pHluorin signal was quenched and PLPPR3 ICDm was uniformly distributed along the axon. Upon neuronal activation with 90 mM KCl solution (Fmax, right), Syph-pHluorin signal becomes visible, indicating synaptic vesicle fusion to the plasma membrane. Scale bars, 5 μm. Boxed areas in each panel are magnified below and were manually traced for quantification. Yellow-highlighted columns in lower graph panels indicate the areas of active SV release identified by increased peaks of pHluorin signal with green solid and dotted lines representing Fmax vs. F0 tracings, respectively. The tracings of mScarlet fluorescence (A and B) and Halo-ICDm proteins (C and D) are also indicated in magenta solid and dotted lines, similarly. (E) Synaptic vesicle release measured as increased fluorescence of pH-sensitive EGFP variant (pHluorin) tagged to the luminal region of synaptophysin upon stimulation with KCl (90 mM). *WT* or *Plppr3−/−* neurons were co-transfected with mScarlet-myristoylation tag (for targeting the protein to membranes; magenta), PLPPR3 ICDm-Halo (green), or the PKA phospho-null variant PLPPR3 ICDm S351A-Halo (purple). Each point in the graph represents a single analyzed bouton. Significance was tested using Kruskal-Wallis Test; ∗∗<0.01; ∗∗∗<0.001; ∗∗∗∗<0.0001. Data are represented as mean ± SEM. The central line represents the median, the boxes include 50% of datapoint distribution (from 25th to 75th percentiles), and whiskers extend until 100% in both ends. (F) Working model of PKA-induced S351 PLPPR3 phosphorylation and presynaptic functions of PLPPR3. Created with Biorender.com.

We first co-expressed PLPPR3 ICDm-Halo and Syph-pHluorin in *WT* neurons and, confirmed by live-cell imaging the enrichment of PLPPR3 ICD in synaptic boutons, marked by accumulations of Syph-pHluorin (Figure S6B). We then asked whether endogenous PLPPR3 controls SV release by performing the pHluorin assay in *Plppr3*^−/−^ neurons transfected with a control mScarlet plasmid and comparing the release in *WT* neurons (Figure 5A). KCl depolarization in neurons triggers PKA activation, which, in concert with other kinases, increases transmitter release as well as the gating and trafficking of voltage-gated calcium channels, leading to increased presynaptic activity.^26^^,^^27^^,^^28^ Before stimulation, the Syph-pHluorin signal was quenched by the acidic pH of the vesicle lumen, but following KCl stimulation, the Syph-pHluorin signal increased, indicating synaptic vesicle release and dequenching in the pH-neutral extracellular space (Figures 5A and 5B; note that areas of peak Fmax fluorescence corresponding to active boutons are highlighted in the lower graph panels for each condition). Quantification of pHluorin fluorescence following stimulation with KCl revealed that in *Plppr3*^−/−^ neurons the SV release was increased when compared to *WT* neurons (Figures 5A, 5B, and 5E). This result suggests that endogenous presynaptic PLPPR3 imposes a tonic inhibitory signal for SV release.

Given that the PLPPR3 ICDm recapitulates the localization of PLPPR3 in presynaptic boutons (Figure 4), we then tested whether expression of wild-type or phospho-dead ICDm S351A would reconstitute the SV release defect in *Plppr3*^−/−^ neurons. Notably, in all conditions, KCL stimulation did not change per se the fluorescence intensity of overexpressed proteins in the wild-type neurons or the PLPPR3 knock-out neurons (Figure S6C). While *Plppr3*^−/−^ neurons rescued with PLPPR3 ICDm were indistinguishable from WT, the expression of the PLPPR3 ICDm S351A mutant failed to rescue SV release to WT levels (Figures 5B–5E). These results suggest that PKA-dependent S351 phosphorylation of PLPPR3 controls neurotransmitter release.

## Discussion

In summary, the work presented here proposes PLPPR3 membrane proteins as signaling integrators at neuronal synapses. Our work demonstrates that presynaptic PLPPR3 controls the exocytosis of synaptic vesicles in axonal boutons. Interestingly, we present evidence that this function depends on PKA-targeted phosphorylation of S351 in the PLPPR3 ICD.

### Phosphorylation of the ICD of PLPPR3 as a signaling switch at the interface of membranes and cytosol

Our work identified two dense clusters of high-confidence phosphorylation sites in the 433 amino acid-long PLPPR3 ICD. One of these clusters stretches from amino acids 343–400 and includes on average one phosphorylation site every five amino acids. The second cluster stretches from amino acids 560–575, where on average every third residue is phosphorylated (Figure 2C). On one hand, these phosphorylation clusters overlap with well-conserved regions in PLPPR protein family ICDs, despite the ICDs having significantly different lengths, ranging from ∼50 to ∼400 amino acids, and being poorly conserved overall.^1^ Thus, these regions could be important structural regulatory domains in all PLPPRs. On the other hand, however, few of the actual phosphorylation sites are well conserved in other PLPPRs. For example, PLPPR3 S343 is present in PLPPR1 and PLPPR4, while in PLPPR5, this residue is substituted with the negatively charged glutamic acid; PLPPR3 S565 shows a conservative substitution to threonine in PLPPR4 and is substituted with a negatively charged glutamic acid in PLPPR1 and PLPPR5. Thus, it is likely that these few conserved sites could regulate functions common to all PLPPRs, such as filopodia formation. In contrast, other sites within these phosphorylation clusters and common regions, such as PLPPR3 S351, are less conserved among different PLPPRs, while showing high conservation in PLPPR3 across different species (Figure 2B), suggesting these sites regulate functions unique to PLPPR3. This is in good agreement with our data showing that S351 does not regulate filopodia formation (Figure S1).

What could be the function of such many phosphorylation sites in PLPPR3-ICD? It is particularly interesting that the polyE box consisting of a stretch of 20 negatively charged glutamic acids is located between the two densely phosphorylated clusters (Figure 2C). It is conceivable that these clusters, together with the polyE box, may regulate the positioning of the ICD in relation to the inner plasma membrane. Indeed, our unpublished data indicate that PLPPR3 binds membrane phosphoinositides via its ICD, and the same has been published for PLPPR5.^29^ PLPPR3 also binds PTEN,^3^ a protein that interacts with acidic phospholipids in membranes and inhibits its activity via the polyE box (Premeti, Syropoulou, Bintig, Eickholt, Leondaritis, unpublished). Thus, phosphorylation would increase the total negative charge of the PLPPR3-ICD, perhaps repelling ICD from the inner leaflet of plasma membrane, and, simultaneously, making it available for interactions with other membrane proteins or cytosolic proteins attracted to highly acidic residues.

### PKA-dependent phosphorylation of PLPPR3 at S351 induces interaction with BASP1

We focused on PLPPR3 S351 phosphorylation because this site is evolutionarily fully conserved between PLPPR3 from different species (Figure 2B) and has also been identified in independent phosphoproteome studies of brain tissue.^30^^,^^31^^,^^32^ Furthermore, our analysis suggests that it is present in developing primary hippocampal neurons and in adult brain synaptosomal fractions (Figures 4A–4C). Interestingly, a recent phosphoproteomic study showed that PLPPR3 S351 phosphorylation is regulated endogenously *in vivo*. Pinto et al. studied TNFα-dependent phosphorylation of proteins in the cortex during sleep, and, curiously, among others, dynamic regulation of PLPPR3 pS351 was observed.^33^

We rigorously characterized phosphorylation of PLPPR3 S351 as a PKA-dependent and cAMP-induced phosphorylation event (Figures 3A–3E) that regulates binding to BASP1. BASP1 together with related GAP43 is acidic 23–25 kDa intrinsically disordered proteins that have been extensively studied for their role in regulating cortical actin dynamics and availability of plasma membrane phosphatidylinositol-4,5-bisphosphate at the inner leaflet of plasma membrane.^16^^,^^17^ BASP1, specifically, has been demonstrated to regulate axon growth and regeneration.^9^^,^^17^^,^^34^ However, several low- and high-throughput studies have suggested a synaptic function for BASP1. Discovery mass spectrometry studies consistently find BASP1 in the synaptic fractions,^35^^,^^36^^,^^37^^,^^38^ while early electron microscopy studies have identified BASP1 in presynaptic densities and synaptic vesicles.^39^^,^^40^ Furthermore, co-localization and interaction with presynaptic vesicle associated proteins, such as VAMP2, synaptojanin-1, and dynamin-1, suggest a role in synaptic transmission or synaptic vesicle recycling.^41^^,^^42^^,^^43^ Indeed, our experiments show that BASP1, as well as PLPPR3, is localized in axonal presynaptic boutons and that, under basal conditions, the deletion of PLPPR3 results in mislocalization of endogenous BASP1 away from presynaptic VGLUT1 clusters.

### PLPPR3 controls presynaptic vesicle release

Our ongoing studies on PLPPR family proteins have suggested that, contrary to previous assumptions, expression of PLPPR3 (and other PLPPRs) ensues in the adult brain.^1^^,^^5^ Furthermore, in different adult rodent brain areas, PLPPR3 is recovered almost exclusively in synaptosomes (Figure 4C).^5^ In early development, where it is most highly expressed, endogenous PLPPR3 localizes to axons, specifically to axonal filopodia and branches.^2^^,^^3^^,^^4^ During this developmental window, PLPPR3 is shown to be essential for LPA-dependent axon guidance *in vivo* and generation of axonal filopodia and branches *in vitro*.^2^^,^^3^^,^^4^ However, in later developmental stages, as in DIV16 hippocampal neurons, decline of overall expression of PLPPR3 coincides with a restricted localization in presynaptic axonal boutons labeled by endogenous VGLUT1 and overexpressed Synaptophysin1, standard markers of nascent and mature axonal boutons (Figures 4D and E). It is noteworthy that phosphorylation of endogenous PLPPR3 S351 was most potently triggered by Forskolin-induced PKA activation at 9 DIV (Figure S3B), which is when cultured neurons undergo synaptogenesis (generally considered 7–14 DIV).^44^ We reasoned that this developmental shift of PLPPR3 expression levels and localization, together with the co-expression of PLPPR3 and BASP1 in axonal boutons, might indicate an unknown synaptic role for PLPPR3 and the S351 phosphorylation.

Here, we provided several lines of evidence that this is indeed the case. Firstly, we quantified SV release upon KCl-induced depolarization in mature hippocampal neurons, by using a standard Syph-pHluorin probe. Secondly, we verified that expression of a membrane-tagged version of the isolated PLPPR3-ICD recapitulates the localization of endogenous PLPPR3 in Synaptophysin-positive axonal boutons (Figure 4E). This allowed us to use the isolated ICD and its phospho-ablating S351A mutant to assess the role of S351 phosphorylation in axonal boutons.

Surprisingly, analysis of *Plppr3*^−/−^ hippocampal neurons showed increased SV release, suggesting that endogenous presynaptic PLPPR3 imposes a tonic inhibitory signal for SV release (Figure 5). This tonic inhibitory signal may relate to possible interactions with proteins of the SV secretion machinery or to deregulation of KCl-induced membrane depolarization, for example activity of voltage-gated calcium channels. Alternatively, increased (Fmax-F0)/Fmax upon PLPPR3 depletion could also be due to alteration of SV pools, for example, increased size of the readily releasable pool of SVs. Nevertheless, KCl-induced depolarization in neurons activates PKA, which via phosphorylation of multiple targets including Synapsin-1 and voltage-gated calcium channels leads to increased presynaptic activity.^26^^,^^27^^,^^28^ This model would imply that S351 phosphorylation of PLPPR3 might serve to fine-tune or control the extent of presynaptic potentiation by PKA. In line with a PKA/PLPPR3-dependent mechanism, reconstitution of endogenous PLPPR3 by overexpression of the isolated ICD in *Plppr3*^−/−^ neurons, corrected KCl-induced SV release to *WT* levels (Figure 5). Remarkably, however, overexpression of the S351A phospho-dead mutant of PLPPR3-ICD, despite its robust localization in Synaptophysin1-labeled axonal boutons, failed to rescue KCl-induced SV release in *Plppr3*^−/−^ neurons (Figure 5) but nevertheless supports BASP1 localization to presynaptic boutons (Figure 4). Together, these experiments demonstrate that, firstly, PLPPR3 controls SV release, at least *in vitro*, and secondly, S351 phosphorylation of PLPPR3-ICD is involved in this process. However, the phosphorylation-dependent effects involved in the control of vesicles release may occur independently of BASP1 recruitment.

### A role for a G protein-coupled receptors (GPCR)-regulated PLPPR3 at the CNS synapse

A limitation of our study relates to the *in vitro* experiments in mature hippocampal neurons, which used a generic high concentration KCl protocol for triggering presynaptic activity. This approach is comparatively strong compared to common physiological stimuli, suggesting a variable dependency of different modes of SV release on presynaptic PLPPR3. Adding to this, recent studies indicate ensuing but variable expression of PLPPR3 in certain GABAergic and glutamatergic neuronal subtypes in the cortex and the hippocampus,^5^ while PLPPR3 is expressed also in adult synaptosomes of the prefrontal cortex and striatum.^5^ As such, it is likely that this presynaptic role of PLPPR3 in regulating synaptic vesicle release may relate to specific neuronal networks in the brain. Our work provides the groundwork for future studies that could directly assess PLPPR3 role in synaptic functions of specific neuronal networks (in terms of topology and synapse type) using electrophysiological methods as well as behavioral assays. Furthermore. the PLPPR3 S351 antibody that we developed here could be used as a tool to probe the involvement of GPCR-PLPPR3 association in CNS disease mouse models.

All of the above raise exciting possibilities of fine spatiotemporal regulation of PLPPR3 phosphorylation at the presynapse by many neurotransmitters that activate GPCRs coupled with Gs or Gi/o proteins and, hence, regulate PKA activity. Adenosine, serotonin, endocannabinoids, and dopamine all regulate presynaptic GPCR receptors in the brain.^45^^,^^46^^,^^47^^,^^48^^,^^49^ Future studies will need to investigate potential regulation of the PKA-cAMP-driven phosphorylation of PLPPR3 S351 and the possible interaction with BASP1 in axonal terminals by different neurotransmitter systems *in vivo*.

### Limitations of the study

As stated above, our *in vitro* experiments have used a generic high concentration KCl protocol for triggering presynaptic activity and Forskolin treatments for activating PKA, which are comparatively strong compared to common physiological stimuli.

## Resource availability

### Lead contact

Requests for resources and reagents should be directed to the lead contact, Britta J. Eickholt (britta.eickholt@charite.de).

### Materials availability

All described reagents this study generated, i.e., plasmids and mouse lines, are available from the lead contact.

### Data and code availability

• Raw data of western blots, PhosTag blots, and MS data for this paper were deposited on Mendeley at https://doi.org/10.17632/w97nrdvxbc.1. (https://doi.org/10.17632/w97nrdvxbc.1).
• The python script for cluster distance measurement is available on GitHub (https://github.com/ngimber/axonal_cluster_workflow). Critical analysis tools used in the study are specified in the key resources table as well as the method details section.
• Any additional information is available from the lead contact upon request.

## Acknowledgments

We thank Kerstin Schlawe and Kristin Lehmann for excellent technical assistance. We thank Katrin Büttner for help with cloning Susanne Wegmann and Janine Hochmair (DZNE Berlin) for help with kinase assays. We thank Joachim Fuchs, Till Mack, and Patricia Kreis (Charité Universitätsmedizin Berlin) for helpful discussions and Alexandra Polyzou (University of Ioannina, Leondaritis lab) for sharing her unpublished data with us. We are grateful to the Advanced Medical Bioimaging (AMBIO) Core Facility as well as to the NeuroCure Multi-user Microscopy Core Facility of Charité for assistance on microscopy and image analysis and to Pico Caroni for sharing antibodies with us. Dorothee Günzel and Jörg Piontek are gratefully acknowledged for their technical support and expertise with STED microscopy. Parts of this manuscript were published as a monograph to obtain the doctoral degree (available at https://refubium.fu-berlin.de/handle/fub188/41517). This work was funded by the 10.13039/501100013209Hellenic Foundation for Research and Innovation to G.L. (H.F.R.I.; “2nd Call for H.F.R.I. Research Projects to support Faculty Members & Researchers” project 02667), the DFG Research Unit 5228 to B.J.E. (Project A2), the DFG Collaborative Research Centre SFB958 (Project A16) to B.J.E. and (Project Z02) to J.S., the SFB1286 (Project B10) to D.M., and the DFG Grant MI 2104 to D.M.

## Author contributions

Conceptualization: B.J.E., G.L., C.K., and D.M.; data curation: M.K., N.G., K.T.T., G.A.P., C.K., and D.N.H.; formal analysis: C.K., G.A.P., D.N.H., M.K., N.G., and K.T.T.; funding acquisition: B.J.E., G.L., D.M., J.S., and P.M.; investigation: C.K., S.B., G.A.P., D.N.H., D.R., V.S., M.K., W.B., A.B., G.B., and T.A.Z.; methodology: C.K., S.B., G.A.P., N.G., and B.J.E.; project administration: C.K. and B.J.E.; resources: B.J.E., C.K., and S.C.; software: N.G.; supervision: B.J.E.; validation: C.K., G.A.P., and B.J.E.; visualization: C.K., G.A.P., D.N.H., V.S., and B.J.E.; writing – original draft: C.K., B.J.E., G.L., G.A.P., and D.M.; and writing – review & editing: C.K., S.B., G.A.P., D.N.H., D.R., V.S., S.C., M.K., N.G., W.B., A.B., G.B., K.T.T., T.A.Z., P.M., J.S., D.M., G.L., and B.J.E.

## Declaration of interests

The authors declare no competing interests.

## STAR★Methods

### Key resources table

**Table:** 

REAGENT or RESOURCE	SOURCE	IDENTIFIER
**Antibodies**		

PLPPR3 (1.55 μg/μl)	custom made^3^ (Eurogentec)	N/A
PLPPR3 pS351 (2.4 μg/μl)	custom made (Eurogentec)	N/A
BASP1 (0.25 μg/μl)	Developmental sample (Cell Signaling Technology)	N/A
DARPP32 pT34	Phosphosolutions	Cat#P1025-34; RRID: AB_2492068
Akt	Cell Signaling Technologies	Cat#9272; RRID: AB_329827
Akt pT450	Cell Signaling Technologies	Cat#9267; RRID: AB_823676
α-tubulin	Sigma-Aldrich	Cat#T6199; RRID: AB_477583
GFP	Genetex	Cat#GTX13970; RRID: AB_371416
GluA1 pS845 (clone D10G5)	Cell Signaling Technologies	Cat#8084; RRID: AB_10860773
VGLUT1	Synaptic Systems	Cat#135303; RRID: AB_887875
Flag	Sigma-Aldrich	Cat#F1804; RRID: AB_262044
Synaptophysin-1 (clone SVP-38)	Sigma-Aldrich	Cat#S5768; RRID: AB_477523
PSD-95	Antibodies Incorporated	Cat#75-028; RRID: AB_2292909
turbo GFP (clone2H8)	Origene	Cat#TA150041; RRID: AB_2622256
Alexa Fluor 647 phalloidin		Cat#A22287
α-rabbit IgG-HRP	Invitrogen	Cat#PI-1000; RRID: AB_2916034
α-mouse IgG-HRP	Vector Laboratories	Cat#PI-2000; RRID: AB_2336177
α-chicken-Alexa488	Vector Laboratories	Cat#703-545-155; RRID: AB_2340375
α-mouse- DyLight550	Jackson ImmunoResearch	Cat#NBP1-75616; RRID: AB_11027384
α-rabbit-Alexa488	Novus Biologicals	Cat#711-545-152; RRID: AB_2313584
α-rabbit-Cy3	Jackson ImmunoResearch	Cat#711-165-152; RRID: AB_2307443
α-guinea pig-Alexa647	Jackson ImmunoResearch	Cat#706-605-148; RRID: AB_2340476
mouse IgG	Jackson ImmunoResearch	Cat#015-000-003; RRID: AB_2337188

**Biological samples**		

Mouse: Acute brain slices (*WT* C57/BL6)	The Jackson Laboratory	Cat#000664; RRID: IMSR_JAX:000664

**Chemicals, peptides, and recombinant proteins**		

Forskolin	Cayman Chemical	Cat#11018
8-Br-cAMP	Sigma-Aldrich	Cat#B7880
H89	Tocris	Cat#2910
Cantharidin	Roth	Cat#3322.1
Protease inhibitor cocktail set III	Calbiochem	Cat#539134
Lamda phosphatase	NEB	Cat#P0753
AEBSF	Applichem	Cat#1421
PKA catalytic subunit	Biolabs	Cat#P6000S
PhosTag reagent	Thermo Scientific	Cat#AAL-107
Protease inhibitor tablets	Roche	Cat#4693159001
Fos-choline 14	Anatrace	Cat#F312
Phospo-peptide: LKRApSVDVDLLA	Eurogentec	N/A
Non-phospho-peptide: LKRASVDVDLLA	Eurogentec	N/A
Kynurenic acid	Sigma	Cat#K3375

**Critical commercial assays**		

Pierce BCA Protein Assay Kit	Thermo Scientific	Cat#23225
Sulfolink column	Thermo Scientific	Cat#44999

**Deposited data**		

Raw data i.e. western blots, phostag blots and MS data	This paper; Mendeley	Mendeley Data: https://doi.org/10.17632/w97nrdvxbc.1
Plasmid generation protocol	This paper; protocols io	https://doi.org/10.17504/protocols.io.dm6gp37j8vzp/v1

**Experimental models: Cell lines**		

Human: HEK293T	BioCAT/SBI	Cat#LV900A-1; RRID: CVCL_UL49
Mouse: N1E-115	ATCC	Cat#CRL-2263; RRID: CVCL_0451

**Experimental models: Organisms/strains**		

Mouse: Primary hippocampal and cortical neurons *(C57 Bl/6NCrl*)	The Jackson Laboratory	Cat#000664; RRID: IMSR_JAX:000664
Mouse: Primary hippocampal and cortical neurons *(C57 Bl/6NCrl PTEN*^*fl/*^*^fl^)*	Trotman et al., 2023^50^	N/A
Mouse: Primary hippocampal and cortical neurons (*C57 Bl/6NCrl Plppr3*^*-/-*^)	Brosig et al., 2019^3^	N/A

**Oligonucleotides**		

Primer: ICDm-His; Forward: aattcgCTAGCgccaccAtgggc tgcgtgcagtgcaaagataaagaagcgcaggcaccacctgca; Reverse: ggtggtgcctgcgcttctttatctttgcactgcacgcagccca TggtggcGCTAGcg	This paper	N/A
Primer: ICDm-S351A-His; Mutagenesis Forward: CTGAAGCGAGCCgcCGTGGATGTGGAC; Mutagenesis Reverse GTCCACATCCACGgcGGCTCGCTTCAG; Extension Forward and Reverse: same as ICDm-His	This paper	N/A
Primer: ICDc-His; Forward: aattcgCTAGCgccaccAtgg CGtgcgtgcagtgcaaagataaagaagcgcaggcaccacctgca; Reverse: ggtggtgcctgcgcttctttatctttgcactgcacgcaCGccaT ggtggcGCTAGcg	This paper	N/A
Primer: BASP1-Flag; Forward: tagagCTAGCgccacc ATGGGAGGCAAGCTGAGC; Reverse: ccaccggatcc CTCTTTGACGGCCACGCTTTGCTCGGAG	This paper	N/A
Primers all other constructs, see STAR Methods: plasmids section	N/A	N/A

**Recombinant DNA**		

Plasmid: ICDm-His	This paper	N/A
Plasmid: ICDm-S351A-His	This paper	N/A
Plasmid: ICDc-His	This paper	N/A
Plasmid: N-S351A-Flag	This paper	N/A
Plasmid: N-S379A-Flag	This paper	N/A
Plasmid: N-T380A-Flag	This paper	N/A
Plasmid: N-S379A/T380A-Flag	This paper	N/A
Plasmid: N-S351A/S379A/T380A-Flag	This paper	N/A
Plasmid: PLPPR3-S351A-Flag	This paper	N/A
Plasmid: PLPPR3-S351D-Flag	This paper	N/A
Plasmid: BASP1-Flag	This paper	N/A
pPAL_ICD-His	Fatih Ipek	N/A
pCAX_N-Flag	Dr. Joachim Fuchs/Dr. George Leondaritis^3^	N/A
pCAX_Cm-Flag		N/A
pCAX_Cc-Flag		N/A
pCMV6_BASP1-tGFP	Origene	Cat#MG217147
pCAX_PLPPR3-Flag	Brosig et al.^3^	N/A
pN1_GFP-F	Jiang and Hunter^51^	N/A
f(syn)-Syp-GFP-w	Viral Core Facility, Charité^52^	N/A

**Software and algorithms**		

NIS Elements	Nikon	N/A
MaxQuant v1.6.0.1 and v1.6.3.4	Tyanova et al., 2016^53^	N/A
ImageJ	Schneider et al., 2012^54^	https://imagej.net/ij/
Graphpad Prism 9.0.0	GraphPad Software, Boston, Massachusetts USA	N/A
BioRender	N/A	https://www.biorender.com/

**Other**		

NetPhos3.1 Prediction Tool	DTU Health Tech	https://services.healthtech.dtu.dk/services/NetPhos-3.1/
COBALT: Constraint-based Multiple Alignment Tool	NIH	https://www.ncbi.nlm.nih.gov/tools/cobalt/cobalt.cgi?CMD=Web
ImageJ macro for analysis of filopodia density	Fuchs, 2022^19^	https://github.com/jo-fuchs/Filopodia_Membrane_recruitment
Python script for distance measurements	GitHub, Niclas Gimber	https://github.com/ngimber/axonal_cluster_workflow

### Experimental model and study participant details

#### Animal procedures

Animals were handled and housed in accordance with the local ethical guidelines and regulations. The animals were kept under standard conditions with a 12-hour light/dark cycle and food and water available at all times. All animal experiments were registered in the Landesamt für Gesundheit und Soziales (LaGeSo) under license T0347/11. All animals used in this work were with the C57 Bl/6NCrl genetic background. Primary cultures from wild-type and *PTEN*^*fl/fl*^ animals were used as control for the *Plppr3*^*-/-*^.^3^^,^^50^ No experiments were analyzed by sex.

#### Cell line culturing and transfection

HEK293T (#LV900A-1, BioCAT/SBI, RRID: CVCL_UL49) and N1E-115 (#CRL-2263, ATCC, RRID: CVCL_0451) cells were cultured in DMEM high glucose (#11965092, Life Technologies) supplemented with 10% fetal bovine serum (#F7524, Sigma; heat inactivated) and 1% penicillin/streptomycin (#15140122, Gibco) at 37°C with 5% CO_2_. Cells were passaged twice a week at a ratio of 1:4.

HEK293T cells were plated on poly-DL-ornithine (#P8638, Sigma; 15 μg/ml for 1 hour at 37°C) coated glass coverslips or plastic cell culture dishes. N1E-115 cells were grown without substrate. For western blot and immunoprecipitation experiments, cells were plated at a density of 300 000/well on 6-well culture dishes (#92006, TPP). For immunocytochemistry experiments, cells were plated at a density of 20 000 (HEK293T) or 30 000 (N1E-115) per well on 12-well culture plates (#92012, TPP). Cells were grown overnight before transfection.

Cell lines were transfected with Lipofectamine 2000 (#11668019, Invitrogen) in Opti-MEM reduced serum medium (#31985062, Gibco), always using 1 μg of total DNA and 2 μl of Lipofectamine per well. For 12-wells, 300 μl of Opti-MEM/well was used, for 6-well plates 400 μl/well was used. Transfection mix was prepared in two steps. First, half of the Opti-MEM volume was mixed with DNA, and the other half with Lipofectamine, mixed, and incubated at room temperature for 5 minutes. Then, the DNA and Lipofectamine were pooled and incubated on the water bath at 37°C for 10 minutes. Transfection mix was added dropwise directly to the cell culture medium and incubated for 5 hours. Finally, the medium was aspirated and exchanged with fresh complete medium. For experiments that required starvation, cells were changed into FBS-free medium consisting only of DMEM and penicillin/streptomycin. Cells were grown overnight before experimental procedures.

#### Primary neuronal culture and transfection

To prepare primary neuronal cultures, cortices and hippocampi of embryos (both female and male) from E16.5 pregnant mice were dissected into individual Eppendorf tubes containing cold HBSS (2 hippocampi/tube, one hemisphere of cortex/tube). The tissue was rinsed with fresh HBSS and digested with 20 U/ml papain (#LS003126, Worthington Biochemical) (500 μl per one hippocampi Eppendorf, 1 ml for cortex) for 30 minutes on thermomixer at 37°C with gentle shaking (350 rpm). Papain digestion was stopped by replacing the supernatant with prewarmed inactivation solution [DMEM with 10% FCS, 1% Pen/Strep, 2.5 mg/ml Albumin/BSA (#A2153, Sigma) and 2.5 mg/ml trypsin inhibitor (#T9253, Sigma)] and incubating at 37°C for 5 minutes. Then, supernatant was replaced with 200 μl plating medium (Neurobasal, 10% heat inactivated FCS, 2% B27, 1% GlutaMax and 1% Pen/Strep) and tissue was disrupted by gentle trituration. Neurons from different preparation tubes were pooled and counted.

Neurons were plated in plating medium and after 2 hours changed to complete medium (Neurobasal, 2% B27, 0.5% Glutamax and 1% Pen/strep). For western blot experiments, cortical or hippocampal neurons were plated on poly-DL-ornithine (15 μg/ml) coated plastic 6-well dishes at a density of 500 000/well. For immunocytochemistry experiments, hippocampal neurons were plated on poly-DL-ornithine (15 μg/ml) + laminin (20 μg/ml; #L2020, Sigma-Aldrich) coated 18 mm glass coverslips at a density of 120 000/well. Primary cultures were grown under constant culture conditions (37°C, 5% CO_2_) until further use.

For immunocytochemistry experiments on Figures 5B–5F, hippocampal neurons were transfected at DIV1 using the calcium phosphate method.^55^ In total, 4 μg of DNA was used per well (2+2 μg/well for double transfections). To prepare the transfection solution, CaCl_2_ (2.5 μl/well) was diluted in H_2_O (22.5 μl/well) and briefly mixed, then DNA was added and mixed. Then, 2xBSS buffer (25 μl/well; 50 mM BES, 280 mM NaCl, 1.5 mM Na_2_HPO_4_, pH 7.26) was added dropwise and the solution was gently mixed by tapping. Finally, pre-warmed Neurobasal medium with 2% B27 and 0.25% GlutaMax (450 μl/well) was added, and the transfection mix was incubated for 15 minutes at 37°C. Conditioned culture medium was collected from each well and exchanged with transfection medium, and neurons were incubated for 20 minutes. Each well was washed three times with 500 μl warm HBSS buffer (135 mM NaCl, 4 mM KCl, 1 mM Na_2_HPO_4_, 2 mM CaCl_2_, 1 mM MgCl_2_, 20 mM HEPES, 20 mM d-Glucose, pH 7.3). After the final wash, warm conditioned medium was added back to wells. Neurons were grown until further use.

### Method details

#### Cloning

All plasmids generated for this work are in pCAX backbone (CAG promoter) and have a C-terminal tag (Flag tag separated with a flexible GSGGGSG-linker, His tag with a VDGRP-linker). All constructs were validated by control digest and sequencing. A detailed protocol that describes the generation of all the plasmids used in this work can be found at https://doi.org/10.17504/protocols.io.dm6gp37j8vzp/v1. Primers used to generate constructs listed in the key resources table are available in STAR Methods: plasmids section.

#### Acute brain slice preparation and stimulation with Forskolin

*WT* C57/BL6 mice were deeply anesthetized with isoflurane and then decapitated. The brains were quickly cooled in ice cold solution (110 mM choline chloride, 2.5 mM KCl, 1.25 mM NaH_2_PO_4_, 26 mM NaHCO_3_, 11.6 mM sodium ascorbate, 3.1 mM sodium pyruvate, 7 mM MgCl_2_, 0.5 mM CaCl_2_ and 10 mM d-glucose, pH 7.4) and sectioned into 300 μm thick coronal slices on a vibrotome. The acute brain slices were then incubated in the same solution at 32°C for 5 minutes, followed by incubation in artificial cerebrospinal fluid (ACSF; 125 mM NaCl, 2.5 mM KCl, 1.25 mM NaH_2_PO_4_, 25 mM NaHCO_3_, 2 mM CaCl_2_, 1 mM MgCl_2_, and 25 mM dextrose) at 32°C for 30 minutes prior to use. Each brain slice was further dissected, and three half slices were used for each experimental condition. Test slices were placed into an incubation chamber containing ACSF with Forskolin (30 μM) for 15 minutes at 32°C. Control slices remained in an incubation chamber with ACSF lacking Forskolin. Following Forskolin stimulation, test and control brain slices were placed in separate Eppendorf tubes and snap frozen in liquid nitrogen and stored at -80°C until further use. All solutions were saturated with 95% O_2_/5% CO_2_.

#### Drug treatments: DMSO, Forskolin, H89, 8-Br-cAMP

All drugs were added directly into cell culture medium and mixed by gentle trituration. For acute brain slice treatment, Forskolin was added into 100 ml ACSF, and the mixture was allowed to homogenize by air flow for a few minutes before brain slices were added. For experiments requiring DMSO control treatments, at least one control treatment was carried out per experiment to ensure the solvent has no effect. Forskolin (#11018, Cayman Chemical) was used at a final concentration of 30 μM for 5-15 minutes, depending on the experiment (see figure descriptions). 8-Br-cAMP (#B7880, Sigma-Aldrich) was used at 0.5-1 mM concentration for 5 minutes. H89 (#2910, Tocris) was used at 1-50 μM concentration for 1 hour prior to Forskolin treatment.

#### Cell and tissue lysis

To lyse cells and neurons, culture medium was aspirated, and cells were rinsed with ice cold PBS (prepared from tablets, #A9191, Applichem). PBS was aspirated and lysis buffer (200 μl for neurons, 300 μl for cell lines) was added, cells were scraped off the surface and collected into an Eppendorf tube. Cells were incubated in lysis buffer at 4°C for 20 minutes with overhead rotation, followed by centrifugation at 14 000 rpm for 20 minutes at 4°C. Supernatant was collected into a fresh tube, and samples were either used directly or kept in -20°C until use.

To prepare brain lysates used in Figure 4D, whole adult mouse brain (ca 0.45 g) was mechanically disrupted in 4.5 ml lysis buffer using Dounce homogenizer. To prepare brain slice lysates used in Figure 4C, the slices were homogenized in 1 ml lysis buffer using micro tissue homogenizer and trituration with pipet. In both cases, tissue homogenates were incubated in lysis buffer at 4°C for 20 minutes with overhead rotation and centrifuged at 14 000 rpm for 20 minutes at 4°C. Supernatant was collected into a fresh tube, and samples were either used directly or kept in -20°C until use.

All samples were lysed in house-made Ripa buffer (50 mM Tris-HCl, 150 mM sodium chloride, 1% NP-40, 0.5% sodium deoxycholate and 0.1% sodium dodecyl sulfate, pH 8.0) supplemented with lab-made phosphatase inhibitors [1 mM Na_2_MO_4_, 1 mM NaF, 20 mM β-glycerophosphate, 1 mM Na_3_VO_4_ and 500 nM Cantharidin (#3322.1 Roth)] and commercial protease inhibitors (protease inhibitor cocktail III, #539134, Calbiochem). Phosphatase inhibitors were omitted when Lambda phosphatase treatment was required. All samples used for western blot experiments, except the co-immunoprecipitation samples, were measured for total protein concentration using the Pierce BCA Protein Assay Kit (#23225, Thermo Scientific) following the manufacturer's protocol.

#### Lambda phosphatase treatment

48 μl of neuron or cell lysate was mixed with 6 μl of 10x NEBuffer for Protein MetalloPhosphatases (PMP), 6 μl of 10 mM MnCl and 6 μl (=2400 units) of Lambda phosphatase (#P0753, NEB). Dephosphorylation was carried out at 30°C for 30 minutes and stopped by addition of Roti Load buffer (#K929.1, Roth). Treatment control included H_2_O instead of Lambda phosphatase and was also incubated at 30°C for 30 minutes. Phosphorylated control was diluted with H_2_O to the same final volume of other samples and kept on ice for 30 minutes.

#### *In vitro* phosphorylation assay

*In vitro* phosphorylation assay was carried out using purified PLPPR3 ICD from *E.coli*. Prior to phosphorylation assay, protease inhibitors were added to the purified protein (1:100 AEBSF; #1421, Applichem). 4.1 μl of PLPPR3 ICD (final concentration 0.075 mg/ml) was mixed with 0.2 μl purified PKA catalytic subunit (final concentration 20 000 Units; #P6000S, Biolabs), 0.8 μl phosphorylation buffer (final concentration of 25 mM HEPES, 100 mM NaCl, 5 mM MgCl, 2 mM EGTA, 25 mM DTT), 1 μl ATP (final concentration 1 mM) and 14 μl H_2_O. Phosphorylation was carried out at 30°C for 2 hours. The reaction was stopped by the addition of Roti Load buffer.

#### Co-immunoprecipitation

Cell lysates (prepared as described above) from 2 wells of 300 000 HEK293T cells each were pooled. 30 μl of protein lysate was taken for input control, mixed with Roti Load and boiled for 5 minutes at 95°C .12 μl of turboGFP or IgG antibody (roughly 1:50) was added to the rest of the protein lysate and incubated at 4°C overnight with overhead rotation. Dynabeads Protein A (#10002D, Invitrogen) were washed with 1 ml Ripa buffer three times, and after the final wash reconstituted in their original volume with Ripa. 20 μl of bead slurry was added to each sample. The sample was incubated for 1 hour at 4°C with overhead rotation and washed 4 × 15 minutes with Ripa at 4°C with overhead rotation. Finally, the beads were eluted with 30 μl 1x Roti Load by boiling at 95°C for 3 minutes. This step was repeated twice to maximize yield.

#### Preparation of crude synaptosomes

Crude synaptosomes were prepared from the brains of 5-week-old female and male mice. Brains were dissected to exclude the cerebellum and the olfactory bulb. The brain was weighed and homogenized in 1 ml/100 mg homogenization buffer (final concentration 5 mM Tris, 1 mM EDTA, 0.32 M sucrose, pH 7.4) supplemented with protease and phosphatase inhibitors (see section on cell and tissue lysis). The lysate was cleared of nuclei and cell debris by centrifugation at 1000 *g* for 10 minutes. Supernatant (S1) was collected and centrifuged at 15,000 *g* for 30 minutes, after which the supernatant (S2) was collected. The remaining pellet containing the synaptosomes (with myelin, membranes and mitochondria) was resuspended in homogenization buffer without sucrose (5 mM Tris, 1 mM EDTA, pH 7.4) and centrifuged at 15 000 *g* for 30 minutes. Supernatant was discarded and the pellet (P2) was snap frozen. All work was carried out at 4°C or on ice.

Prior to western blot analysis, S2 and P2 fractions were thawn on ice, and the P2 were resuspended in 150 μl homogenization buffer (roughly equal to the volume of S2 fractions). The samples were incubated on ice with 15 μl of 10% TritonX-100 for 10 minutes to break open synaptosomal membranes. Total protein concentration was measured with BCA assay (see cell and tissue lysis) and 25 μg of total protein was loaded on the gel.

#### SDS-PAGE, PhosTag SDS-PAGE and western blotting

We used 20 μg of total protein from N1E-115 cells or 40 μg of total protein from neuronal and tissue lysates for SDS-PAGE western blot analysis. Protein lysates were separated on 8-12% acrylamide gels at 80V for 15 minutes followed by 120 V until the dye front ran out. Proteins were transferred onto 20 μm pore size nitrocellulose membranes (#1620097, Biorad) for 2.5 hours at 400 mA on ice. Membrane was briefly rinsed in dH_2_O, labeled with Ponceau S (#A2935, Applichem) solution according to manufacturer's protocol and de-stained in dH_2_O. The membrane was blocked in 5% skim milk (#T145.2, Carl Roth) in TBS-T (5 mM Tris-HCl, 15 mM NaCl, 0.005% Tween20, pH 7.4) for 1 hour at room temperature and incubated with primary antibodies (see STAR Methods: antibody section) in blocking solution at 4°C overnight. Membrane was washed 3 × 10 minutes in TBS-T and incubated with secondary antibodies (see STAR Methods: antibody section) in blocking solution at room temperature for 1 hour, followed by 3 × 10 minutes washes in TBS-T. Blots were developed with ECL Western Blotting Substrate (#W1001, Promega) or ECL Select Western Blotting Detection Reagent (#RPN2235, Cytiva) according to manufacturer's protocol. Images of western blots were acquired with Fusion SL camera (VilberLourmat, Germany) and manufacturer's software using automatic exposure mode. Chemiluminescent signal image and molecular weight marker image were automatically overlaid by the software. For all SDS-PAGE experiments presented in this work, molecular weight marker ranging from 10 to 250 kDa was used (#26620, Thermo Scientific).

For phosphorylation analysis, 8% 50 μM Zn^2+^ PhosTag acrylamide (#AAL-107, FUJIFILM) gels were prepared according to manufacturer's protocol. 40 μg of total protein was loaded and gel electrophoresis was run under a constant current (30 mA/gel) until the dye front ran out. Gels were washed 3 × 10 minutes in transfer buffer containing 10 mmol/L EDTA, and 1 × 10 minutes in transfer buffer without EDTA prior to transfer. Proteins were transferred onto a PVDF membrane with 0.2 μm pore size (#88520, Thermo Scientific) for 2.5 hours at 400 mA on ice. The rest of the steps were carried out as described above. PhosTag blots do not contain molecular weight markers as they do not give meaningful information about the size of proteins and may cause distortion of protein bands (see PhosTag SDS-PAGE guidebook, available at http://www.bujnochem.com/wp-content/uploads/2019/09/FUJIFILM-Wako_Phos-tag-R.pdf). All western blot experiments, with the exception of Figures 4A–4C, were performed a minimum of three times with different passages of cells.

#### Purification of PLPPR3 ICD constructs

12x10^6^ HEK293T cells were seeded in 150 cm^2^ Corning flasks (#CLS431465, Merck) and cultured overnight. Cells were transfected with ICDm-His or ICDc-His constructs using 75 μg of total DNA and 225 μl PEI in 5 ml serum free hybridoma medium (#11279023, Gibco), and grown overnight. Cells were washed with ice cold PBS once and scraped off the flask in fresh 10 ml ice cold PBS on ice. Following centrifugation at 4000 rpm for 1 min at 4°C, PBS was aspirated, and the cell pellet was frozen.

Four independent replicates were purified and analyzed in parallel. Frozen pellets were thawn on ice, lysis buffer was added [20 mM Hepes, 150 mM NaCl, 20 mM Imidazole, phosphatase inhibitor cocktail (1:25), Cantharidin (1:50), protease inhibitor tablets (1 tbl/10 ml, #4693159001, Roche), AEBSF (1:100, #A1421, Applichem), pH 7.4] and the pellets were sonicated for 3 × 60 seconds with 6 cycles and 60% power. Lysate was cleared by centrifugation at 21 000 *g* at 4°C for 30 minutes. For the purification of cytosolic ICDc-His, the supernatant was collected and incubated with Ni SepharoseTM 6 Fast Flow (GE) beads (300 μl/sample) overnight at 4°C on a rotator. For the purification of membrane-bound ICDm-His, protein was extracted from the pellet with 0.5% Fos-choline 14 (#F312, Anatrace) at 4°C for 40 minutes and centrifuged at 21 000 *g* for 1 hour at 4°C. Supernatant was collected and incubated with Ni SepharoseTM 6 Fast Flow (GE) beads overnight at 4°C on a rotator. Sample was cleared on Pierce centrifugal columns (#89896, Thermo Scientific) by gravity flow and washed three times with 20 ml wash buffer (wash 1: 20 mM Hepes, 150 mM NaCl, 50 mM Imidazole, pH 8.5; wash 2: 20 mM Hepes, 500 mM NaCl, 50 mM Imidazole, pH 8.5; wash 3: 20 mM Hepes, 150 mM NaCl, pH 8.5). Finally, beads were solubilized in 800 μl of buffer (20 mM Hepes, 150 mM NaCl, pH 8.5). The buffer was discarded and dry beads with protein were handed to the mass spectrometry facility. Phosphorylation status of samples was confirmed by PhosTag SDS-PAGE prior to mass spectrometry analysis.

#### Affinity chromatography for the identification of PLPPR3 pS351 interaction partners

Sulfolink columns (#44999, Thermo Scientific) were coupled with PLPPR3 peptides constituting the S351 phosphorylation site (phospho: LKRApSVDVDLLA; non-phospho: LKRASVDVDLLA) according to manufacturer’s protocol. Affinity purification was carried out following the manufacturer's protocol using gravity flow instead of centrifugation. Affinity columns were equilibrated to room temperature for 15 minutes before use and equilibrated three times with 2 ml of PBS. Columns were incubated with 400 μl of freshly prepared brain lysate (see section on cell and tissue lysis) in 1600 μl PBS for 1.5 hours at room temperature with overhead rotation. Columns were washed with 2 ml PBS four times and eluted with 2 ml of elution buffer (0.2 M glycine-HCl, pH 2.5) in ∼500 μl fractions into a fresh tube containing 100 μl neutralization buffer (1 M Tris-HCl, pH 8.5). The protein concentration in each fraction was measured by Nanodrop, and fractions with highest protein content were pooled to concentrate protein by ethanol (EtOH) precipitation. For EtOH precipitation, 9 volumes of ice cold 100% ethanol were added to 1 volume of protein solution. To aid precipitation, 2 μl of Glycoblue (#AM9515, Invitrogen) was added to the mix, and the protein was precipitated overnight at 4°C. The following day, the precipitation solution was centrifuged at 14 000 rpm for 1 hour at 4°C, the aqueous solution was discarded, and the protein pellet resolubilized in 100 μl Roti Load buffer and boiled at 95°C for 5 minutes. The protein samples were run on SDS-PAGE, pieces of gel were excised and analyzed by mass spectrometry. Four biological replicates consisting of brain lysates from four individual mice were performed.

#### Phospho-mass spectrometry analysis

Prior to mass spectrometry analysis, beads were resuspended in 20 μl urea buffer (2M urea, 50 mM ammonium bicarbonate, pH 8.0), reduced in 12 mM dithiothreitol at 25°C for 30 minutes, followed by alkylation with 40 mM chloroacetamide at 25°C for 20 minutes. Samples were digested with 0.5 μg Trypsin/LysC, Trypsin/GluC or GluC overnight at 30°C. The peptide-containing supernatant was collected, and digestion was stopped with 1% formic acid. Peptides were desalted and cleaned up using Stage Tip protocol (Rappsilber et al.^56^). Samples were eluted with 80% acetonitrile/0.1% formic acid, dried using speedvac, resuspended in 3% acetonitrile/0.1% formic acid and analysed by LC-MS/MS. Peptides were separated on a reversed-phase column, a 20 cm fritless silica microcolumn with an inner diameter of 75 μm, packed with ReproSil-Pur C18-AQ 3 μm resin (Dr. Maisch GmbH) using a 90 min gradient with a 250 nl/min flow rate of increasing Buffer B concentration (from 2% to 60%) on a High-Performance Liquid Chromatography (HPLC) system (Thermo Fisher Scientific), ionized with electrospray ionization (ESI) source (Thermo Fisher Scientific) and analyzed on a Thermo Q Exactive Plus instrument. The instrument was run in data dependent mode selecting the top 10 most intense ions in the MS full scans (ranging from 350 to 2000 m/z), using 70 K resolution with a 3×106 ion count target and 50 ms injection time. Tandem MS was performed at a resolution of 17.5 K. The MS2 ion count target was set to 5×104 with a maximum injection time of 250 ms. Only precursors with charge state 2–6 were selected for MS2. The dynamic exclusion duration was set to 30 s with a 10 ppm tolerance around the selected precursor and its isotopes. Raw data were analyzed using MaxQuant software package (v1.6.3.4^53^) using a human UniProt database (HUMAN.2019-07) and PLPPR3 sequence database, containing forward and reverse sequences. The search included variable modifications of serine, threonine, and tyrosine in addition to methionine oxidation, N-terminal acetylation, asparagine and glutamine deamidation. Carbamidomethylation of cysteine was set as a fixed modification. Minimal peptide length was set to seven amino acids and a maximum of 3 missed cleavages was allowed. The FDR was set to 1% for peptide and protein identifications. Phosphosite intensity values were normalized for PLPPR3 protein abundance. Results were filtered for reverse database hits and potential contaminants. Phosphosites found in 3/4 replicates with localization probability >0.75 and good MS2 spectra were considered high confidence.

#### Mass spectrometry analysis for PLPPR3 pS351 interaction partners

Gel pieces were cut out and digested with Trypsin prior to mass spectrometry analysis. Protein samples were concentrated on a trap column (PepMap C18, 5 mm × 300 μm × 5 μm, 100Ǻ, Thermo Fisher Scientific) with 2:98 (v/v) acetonitrile/water containing 0.1% (v/v) trifluoroacetic acid at a flow rate of 20 μl/min for a total of 4 minutes. The samples were analyzed by nanoscale LC-MS/MS using a Q Exactive Plus mass spectrometer coupled with an Ultimate 3000 RSLCnano (Thermo Fisher Scientific). The system contained a 75 μm i.d. × 250 mm nano LC column (Acclaim PepMap C18, 2 μm; 100 Å; Thermo Fisher Scientific). The mobile phase A consisted of 0.1% (v/v) formic acid in H_2_O, while mobile phase B consisted of 80:20 (v/v) acetonitrile/H_2_O containing 0.1% (v/v) formic acid. The samples were eluted using a gradient of mobile phase B (3-53%) in 16 minutes, followed by washing with 98% of phase B and equilibration with starting condition using a flow rate of 300 nl/min. Full MS spectra (350–1,650 m/z) were acquired at a resolution of 70 000 (FWHM), followed by data-dependent MS/MS fragmentation (300–2,000 m/z) of the top 10 precursor ions (dissociation method HCD, resolution 17 500, 1+ charge state excluded, isolation window of 1.6 m/z, NCE of 27%, dd 10s, MS 1e6, MSMS AGC 5e5). Maximum ion injection time was set to 50 ms for MS, and 120 ms for MS/MS scans. Background ions at m/z 391.2843 and 445.1200 act as lock masses. Quantification of proteins was performed with MaxQuant software version 1.6.0.1 using default Andromeda LFQ parameter. Spectra were matched to murine (https://www.uniprot.org/; 17 073 reviewed entries), contaminant, and decoy database. The search included the following modifications: methionine oxidation and N-terminal acetylation (variable), carbamidomethyl cysteine (fixed). The false discovery rate was set to 0.01 for peptide and protein identifications. MS2 identifications were transferred between runs using the “Match between runs” option, in which the maximal retention time window was set to 0.7 min. Protein intensities were normalized using the in-built label-free quantification algorithm. Technical and biological replicates for each condition were defined as groups, label-free quantification intensity values were filtered for minimum value of 2 per group and transformed to log2 scale. Differences in protein levels between samples from unphosphorylated and phosphorylated columns were calculated as log2 fold change in protein intensity. Enriched proteins for each condition were determined by a log2 fold change ≤ 0.05 and ≥ 2.

#### Immunofluorescence

Cells and neurons were fixed with pre-warmed 4% PFA (#1040051000, Merck) PBS for 15 minutes, and rinsed with PBS. Cells were permeabilized and nonspecific binding blocked with PBS containing 0.5% TritonX100 (#T8655, US Biological) and 5% goat serum (#16210-072, Gibco) for 1 hour at room temperature. Primary antibodies (total volume 100 μl/coverslip; see STAR Methods: antibody section) were incubated overnight at 4°C. The next day, coverslips were washed 2 × 10 minutes with PBS 0.1% Tween-20 followed by 2 × 10-minute washes with PBS. Secondary antibodies (total volume 100 μl/coverslip; see STAR Methods: antibody section) were applied for one hour at room temperature, followed by 2 × 10-minute washes with PBS 0.1% Tween-20 and 2 × 10-minute washes with PBS. All antibodies were diluted in PBS containing 0.1% Tween-20 (#655205, Calbiochem) and 5% goat serum. Coverslips were mounted with Prolong Glass Antifade Mountant (#P36984, Invitrogen) and kept at 4°C.

#### STED immunofluorescence and imaging

For two colour STED microscopy, neurons were grown on STED-compatible coverslips (Carl Zeiss, Jena, Germany), fixed with pre-warmed 4% PFA in PBS for 15 minutes and rinsed with PBS. Citrate antigen retrieval was performed for 15 minutes at 92^ο^ C, followed by permeabilization using 0,1% Triton X-100 at room temperature for 10 minutes. Cells were blocked in 5% goat serum for 1 hour at room temperature and incubated with primary antibodies against PLPPR3 (1:200), BASP1 (1:200) and VGLUT1 (1:500), overnight at 4^ο^ C. The next day, cells were washed with PBS and incubated with the secondary antibodies for an 1 h at room temperature and then washed again with PBS. Aberrior STAR RED secondary antibodies were used in 1:200 and SRAT ORANGE in 1:500. All antibodies were diluted in PBS containinsg 5% goat serum. Coverslips were mounted with a mounting medium (Abberior GmbH) heated to 65^ο^C. Two colour STED microscopy was performed on an Abberior STED Facility Line system (Abberior Facility Line, Abberior, GmbH) with a 60x/1,4 NA oil UPlanSApo Olympus Objective. A single focal plane in the centre of neuronal processes was imaged, with a pixel size of 20 nm and a pixel dwell time in 10 μs.

#### Prediction of phosphorylation sites and conservation analysis

Prediction of phosphorylation sites was carried out in NetPhos3.1 server (https://services.healthtech.dtu.dk/services/NetPhos-3.1/). The primary amino acid sequence of PLPPR3 intracellular domain (aa 284-716) was submitted, and phosphorylation of serine, threonine and tyrosine residues was selected for prediction. Only scores >.75 were displayed. To analyze conservation of phosphorylation sites across species, Constraint-Based Multiple Alignment Tool (COBALT, https://www.ncbi.nlm.nih.gov/tools/cobalt/cobalt.cgi?CMD=Web) was used. FASTA sequences of mouse (*Mus musculus*; Q7TPB0-1), human (*Homo sapiens*; Q6T4P5), zebrafish (*Danio rerio*; B0V0Y5), tropical clawed frog (*Xenopus tropicalis*; B1H174), rhesus monkey (*Macaca mulatta*; F7GER3) and chicken (*Gallus gallus*; A0A1D5PYJ6) from Uniprot database (https://www.uniprot.org/) were used. The sequences were aligned with default settings.

### Quantification and statistical analysis

#### Quantification of synaptic vesicle (SV) release

To quantify SV release, primary cortical neurons were transfected on DIV 5-7 using a calcium phosphate transfection protocol, adapted from Jiang & Chen and Jackson et al.^57^^,^^58^ Briefly, transfection mixes containing 2 μg of each DNA construct in 1x TE buffer (10 mM Tris-HCl and 1 mM EDTA, pH 7.3) were supplemented with CaCl_2_ to a final concentration of 250 mM (stock: 2.5 M CaCl_2_ in 10 mM HEPES, pH 7.2) and added dropwise to 2x HEBS buffer (274 mM NaCl, 10 mM KCl, 1.4 mM Na_2_HPO_4_, 10 mM glucose, 42 mM HEPES, pH 7.2) with short, slow vortexing between each addition and incubated for 20 min at room temperature to allow crystals to grow. Coverslips with neurons were transferred from their original dish to a fresh dish containing 1 mL of growth medium supplemented with 4 mM kynurenic acid (Sigma K3375, 20 mM stock solution in NBA) and incubated at 37°C, 5% CO_2_ for 20 min before loading the transfection mixture. Then, the final transfection mix was added to neurons and dishes were incubated at 37°C, 5% CO_2_ for 90 min. Afterwards, medium was replaced by 1 mL of NBA with 4 mM kynurenic acid, supplemented with 2.5 mM HCl as wash medium. Cells were incubated at 37°C, 5% CO_2_, for additional 15 min. Finally, the washed coverslips were placed back in the original culture dish with its own previous conditioned medium.

Our setup design was adapted from Hoffmann et al., 2023^25^ In Brief, images in two channels were taken under an Eclipse Ti Nikon Spinning Disk Confocal CSU-X, equipped with OkoLab Live-cells incubator (37°C, 5% CO_2_), pco.edge, SN:18500826 camera, Andor Revolution SD System (CSU-X) and PL APO 60×/1.4NA oil immersion lens objective. The employed excitation wavelengths were 488 nm for SYPH-pHluorin and 561 nm for mScarlet-myr, Halo-ICDm PLPPR3 (JF549), and Halo-ICDm PLPPR3 S351A (JF549). Coverslips containing transfected neurons (25 mm diameter) were mounted in pre-warmed low-KCl Tyrode solution (150 mM NaCl; 4 mM KCl; 2 mM CaCl_2_; 2 mM MgCl_2_; 10 mM HEPES; 10 mM glucose; pH at 7.4 using NaOH) before imaging them, and then placed on the objective stage. After taking an initial z-stack (F0) with Exposure time 100 ms, the Tyrode solution containing high-KCl (154 mM KCl; 2 mM CaCl_2_; 2 mM MgCl_2_; 10 mM HEPES; 10 mM glucose; pH at 7.4 using NaOH) was added dropwise to the coverslip to obtain a final concentration of 64 mM NaCl and 90 mM KCl. Immediately after adding the correspondent volume, another z-stack was taken at the same position (Fmax). Then, NH_4_Cl was added to the coverslip to a final concentration of 50 mM, and a final z-stack was acquired. Images were acquired using Acquisition software NIS Elements and then analyzed with ImageJ (NIH), taking the maximum projection intensity of selected ROIs for both the low-KCl and high-KCl images. Datapoints calculations were performed ((Fmax – F0)/Fmax), averaged and plotted. For statistics, a non-parametric Kruskal-Wallis test was used; ∗∗<0.01; ∗∗∗<0.001; ∗∗∗∗<0.0001.

#### Quantification of western blot band intensities

Quantification of protein band intensities was carried out in Fiji using the Analyze->Gels command. Composed protein band and molecular weight marker images were used for quantification. Rectangular selection, big enough to include the biggest band, was used to select each protein band. Each lane was plotted using the Plot lanes command, and if necessary, a straight line was drawn to close off the area under the curve. Intensity was measured for each band. Co-immunoprecipitation band (PLPPR3) was normalized to immunoprecipitation band (turboGFP) by dividing the intensity PLPPR3/tGFP. The value of each non-stimulated replicate was normalized to 1, and the value of Forskolin-stimulated sample was expressed as Forskolin/control.

#### Analysis of BASP1 and PLPPR3 clusters in synapses

Images of DIV7 hippocampal neurons were acquired on Leica SP8 inverted confocal with 40x objective. Distal axons of healthy neurons with moderate expression of recombinant proteins were imaged. Channels were acquired sequentially, and acquisition filters were adjusted to avoid crosstalk of fluorophores. Z-stacks with a step size of 0.3 μm covering the entire axon were acquired.

The segmentation of PLPPR3, BASP1, Synaptophysin-1 and Drebrin clusters was performed in an automated manner using a custom-written ImageJ macro (segmentation)^54^ and an additional Python script (distance measures), which can be found on GitHub (https://github.com/ngimber/axonal_cluster_workflow). Axons were manually selected (Figure 4J) or automatically segmented based on Synaptophysin-1 (Figures 4F and S5A–S5C) or MAP-2 (Figure S5D) signal as follows: uneven background was corrected by the “Normalize Local Contrast” function and images were smoothed (Gaussian blur, sigma = 280 nm) before binarization with the Triangle method.^59^ The “Closing” operation was used (1.41 μm) to remove small gaps and linear filamentous structures were enhanced via “Ridge Detection” before converting axons into skeletons with the “Skeletonize” function. Clusters of PLPPR3, BASP1, Synaptophysin-1 (pre-synapse) and Drebrin (post-synapse) were segmented with the following procedure: uneven background was compensated by “Rolling Ball Background Subtraction” before applying a Gaussian Blur (sigma = 140 nm) for noise reduction and smoothing. The “Opening function” was used to remove confocal shot noise and small objects. Clusters were binarized by applying an automated threshold (“Intermodes”)^60^ and connected clusters were split into single clusters using the Watershed algorithm. Only clusters that overlapped with the segmented axons were used for further analysis. Nearest neighbor distributions were calculated per image, based on all cluster centers. PLPPR3 clusters were categorized as “within synapses” (Figure 4F) if the cluster center was located within a radius of 1.4 μm (∼ presynaptic diameter)^61^ around the Synaptophysin-1 cluster center.

#### Filopodia density measurements

N1E-115 cells were transfected with farnesylated GFP (independent membrane marker)^51^ combined with PLPPR3-Flag, PLPPR3-S351A-Flag or PLPPR3-S351D-Flag, and additionally labelled for F-actin (independent filopodia marker). Images of individual, non-overlapping cells were acquired on Leica SP8 inverted confocal with 63x objective. Channels were acquired sequentially, and acquisition filters were adjusted to avoid crosstalk of fluorophores. Z-stacks with a step size of 0.6 μm covering the entire cell were acquired. Filopodia density measurements were performed using an ImageJ macro developed by Joachim Fuchs.^19^ The macro automatically detects the borders of a cell and outlines it, providing a measurement of circumference. Filopodia are detected as intensity peaks along this outline. The analysis was carried out in the membrane marker channel. The ImageJ macro of this analysis is available at (https://github.com/jo-fuchs/Filopodia_Membrane_recruitment). Filopodia density experiment was replicated 3 times with different passages of cells.

#### Statistics and data visualization

Statistical analysis and data visualization was performed with Graphpad Prism 9.0.0. Filopodia densities were compared with one-way ANOVA, changes in co-immunoprecipitation were compared with Wilcoxon test. Figures 2A, 3F, and 5F were generated with Biorender.com.

### Additional resources

#### Antibodies

**Table:** Antibodies used in this work

Antibody	Source	Catalog nr	RRID	Dilution	Application
PLPPR3 (1.55 μg/μl)	custom made^3^ (Eurogentec)	N/A	N/A	1:1000	WB
				1:250	ICC
PLPPR3 pS351 (2.4 μg/μl)	custom made (Eurogentec)	N/A	N/A	1:1000	WB
BASP1 (0.25 μg/μl)	Developmental sample (Cell Signaling Technology)	N/A	N/A	1:500	WB, ICC
DARPP32 pT34	Phosphosolutions	P1025-34	AB_2492068	1:1000	WB
Akt	Cell Signaling Technologies	9272	AB_329827	1:1000	WB
Akt pT450	Cell Signaling Technologies	9267	AB_823676	1:1000	WB
α-tubulin	Sigma-Aldrich	T6199	AB_477583	1:5000	WB
GFP	Genetex	GTX13970	AB_371416	1:1000	ICC
GluA1 pS845 (clone D10G5)	Cell Signaling Technologies	8084	AB_10860773	1:1000	WB
VGLUT1	Synaptic Systems	135303	AB_887875	1:1000	ICC
Flag	Sigma-Aldrich	F1804	AB_262044	1:1000	WB, ICC
Synaptophysin-1 (clone SVP-38)	Sigma-Aldrich	S5768	AB_477523	1:1000	WB
PSD-95	Antibodies Incorporated	75-028	AB_2292909	1:1000	WB
turbo GFP (clone2H8)	Origene	TA150041	AB_2622256	1:1000	WB
				1:50	IP
Alexa Fluor 647 phalloidin	Invitrogen	A22287	-	1:500	ICC
α-rabbit IgG-HRP	Vector Laboratories	PI-1000	AB_2916034	1:5000	WB
α-mouse IgG-HRP	Vector Laboratories	PI-2000	AB_2336177	1:5000	WB
α-chicken-Alexa488	Jackson ImmunoResearch	703-545-155	AB_2340375	1:500	ICC
α-mouse- DyLight550	Novus Biologicals	NBP1-75616	AB_11027384	1:500	ICC
α-rabbit-Alexa488	Jackson ImmunoResearch	711-545-152	AB_2313584	1:500	ICC
α-rabbit-Cy3	Jackson ImmunoResearch	711-165-152	AB_2307443	1:500	ICC
α-guinea pig-Alexa647	Jackson ImmunoResearch	706-605-148	AB_2340476	1:500	ICC
mouse IgG	Jackson ImmunoResearch	015-000-003	AB_2337188	1:50	IP

N/A, not applicable; WB, western blot; ICC, immunocytochemistry; IP, immunoprecipitation.

#### Plasmids

**Table:** Plasmids used in this work

Plasmid	Source or reference	Cloning primers
N-S351A-Flag	This paper	Mutagenesis primers: **fw:** CTGAAGCGAGCCgcCGTGGATGTGGAC **rev:** GTCCACATCCACGgcGGCTCGCTTCAG Extension primers: **fw:** GCTAGCgtcaccATGCTTGCTATG **rev:** gtcgcggccgctTTACTTGTCATCGTCATCC
N-S379A-Flag	This paper	Mutagenesis primers: **fw:** CTGCCCCGGGTCgcCACGCCCTCGCTG **rev:** CAGCGAGGGCGTGgcGACCCGGGGCAG Extension primers: Same as above
N-T380A-Flag	This paper	Mutagenesis primers: **fw:** CCCCGGGTCAGCgCGCCCTCGCTG **rev:** CAGCGAGGGCGcGCTGACCCGGGG Extension primers: Same as above
N-S379A/T380A-Flag	This paper	Mutagenesis primers: **fw:** CTGCCCCGGGTCgcCgCGCCCTCGCTG **rev:** CAGCGAGGGCGcGgcGACCCGGGGCAG Extension primers: Same as above
N-S351A/S379A/T380A-Flag	This paper	Same primers as for S351A and S379A/T380A mutants
PLPPR3-S351A-Flag	This paper	Same primers as for N-S351A-Flag
PLPPR3-S351D-Flag	This paper	Same primers as for N-S351D-Flag
pPAL_ICD-His	Fatih Ipek	N/A
pCAX_N-Flag	Dr. Joachim Fuchs/Dr. George Leondaritis^3^	N/A
pCAX_Cm-Flag		
pCAX_Cc-Flag		
pCMV6_BASP1-tGFP	#MG217147, Origene	N/A
pCAX_PLPPR3-Flag	Brosig et al.^3^	N/A
pN1_GFP-F	Jiang and Hunter^51^	N/A
f(syn)-Syp-GFP-w	Viral Core Facility, Charité^52^	N/A

ICD, intracellular domain; Fw, forward; rev, reverse; N/A, not applicable.
