# Supplementary material for: Phosphorylation of presynaptic PLPPR3 controls synaptic vesicle release
Source: iScience. 2025 Aug 27;28(9):113435. doi: 10.1016/j.isci.2025.113435 (PMC12496174; doi:10.1016/j.isci.2025.113435)
Supplement: Document S1. Figures S1–S6 [file mmc1.pdf]

iScience, Volume *28*

Supplemental information

Phosphorylation of presynaptic

PLPPR3 controls synaptic vesicle release

Cristina Kroon, Shannon Bareesel, Gerard Aguilar Perez, Domonkos Nagy-Herczeg, Dimitra Ranti, Vasiliki Syropoulou, Sandra Coveney, Marieluise Kirchner, Niclas Gimber, Willem Bintig, Annika Brosig, Georg Braune, Kathrin Textoris-Taube, Timothy A. Zolnik, Philipp Mertins, Jan Schmoranzer, Dragomir Milovanovic, George Leondaritis, and Britta J. Eickholt

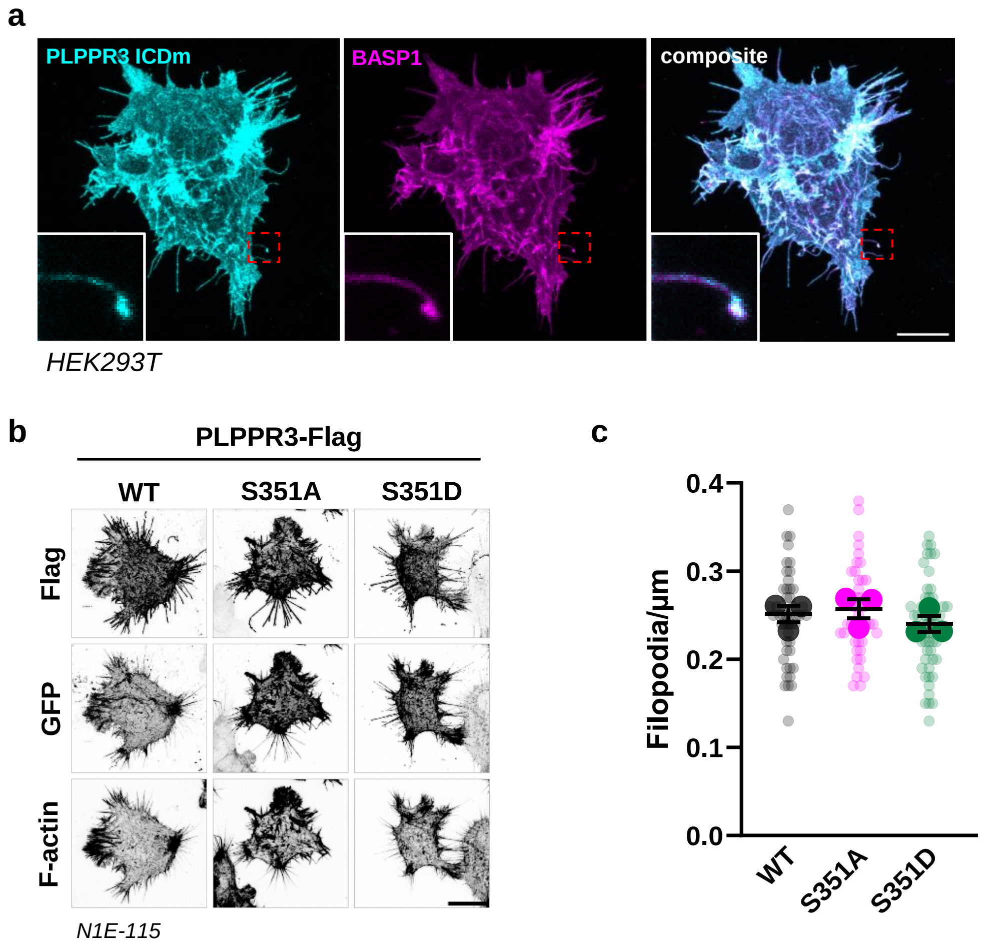

Figure S1. PLPPR3 phosphorylation at S351 does not alter filopodia density. Related to Figure 3.

(a) Co-localization of transfected PLPPR3 ICDm and BASP1 in plasma membrane and filopodia of HEK293T cells. (b) N1E-115 cells were co-transfected with indicated PLPPR3 variants (PLPPR3 WT, PLPPR3 S351A or PLPPR3 S351D) and membrane-tagged GFP, and filopodia density was measured using a semi-automated method. Scale bar = 20 µm. (c) Quantification of experiments. N=3, n=43-52, mean ± SEM.

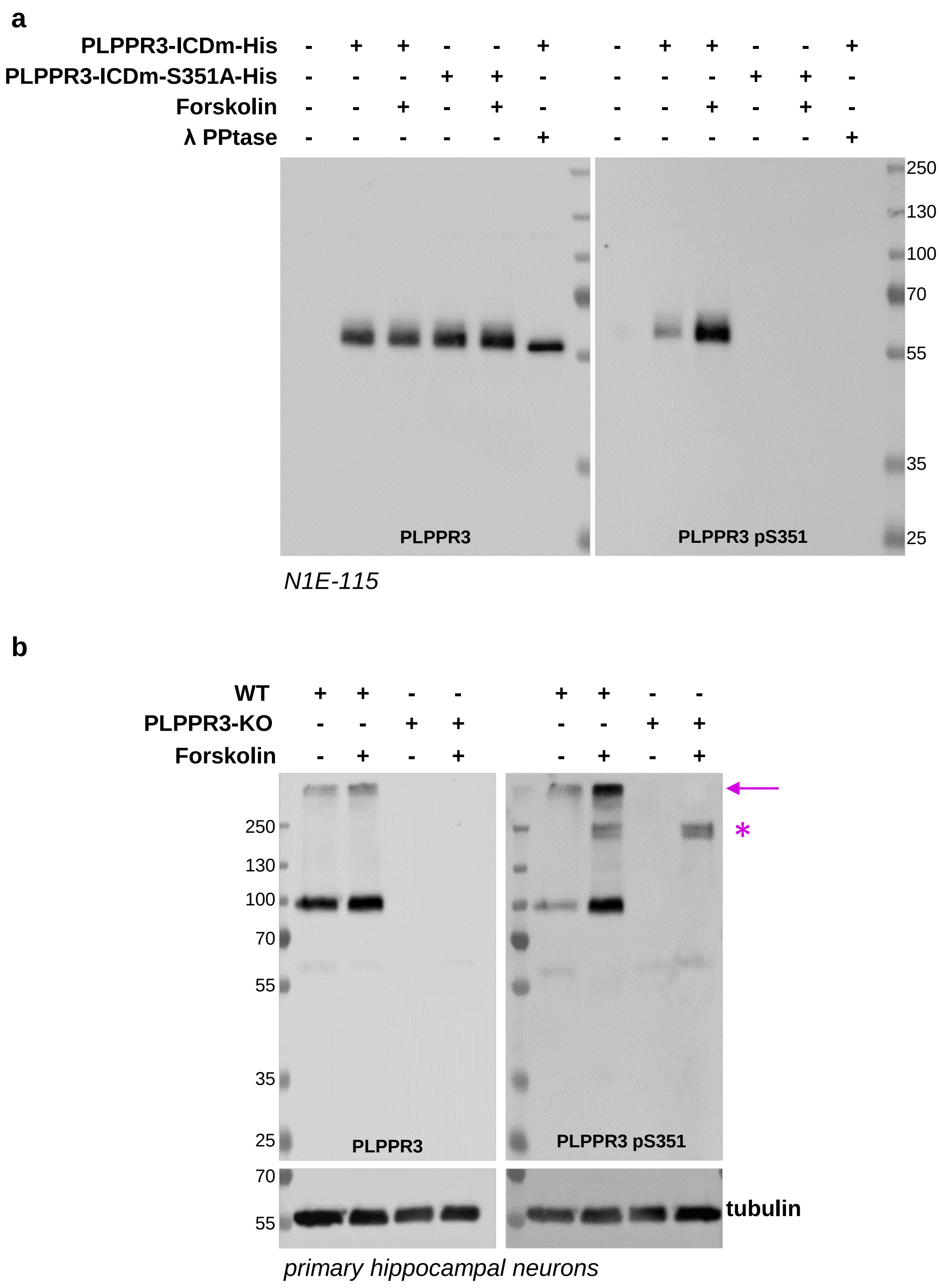

Figure S2. Validation of the custom made PLPPR3 pS351 antibody. Related to Figure 4. (a) Specificity of the PLPPR3 pS351 antibody towards recombinant PLPPR3 ICDm. PLPPR3 ICDm variants were expressed in N1E-115 cells, proteins were extracted, and the lysates were analyzed by western blot. Forskolin stimulation (30 µM, 5 minutes) was performed before lysis. λ phosphatase treatment was performed at 30°C for 30 minutes on phosphatase inhibitor-free samples after cell lysis. (b) Specificity of the PLPPR3 pS351 antibody towards endogenous PLPPR3. Primary hippocampal neurons were lysed at DIV9, proteins were extracted, and the lysates were analyzed by western blot. Forskolin stimulation (30 µM, 5 minutes) was performed before lysis. Unspecific bands are annotated with an asterisk. Higher-order oligomerized forms of PLPPR3 are annotated with an arrow.

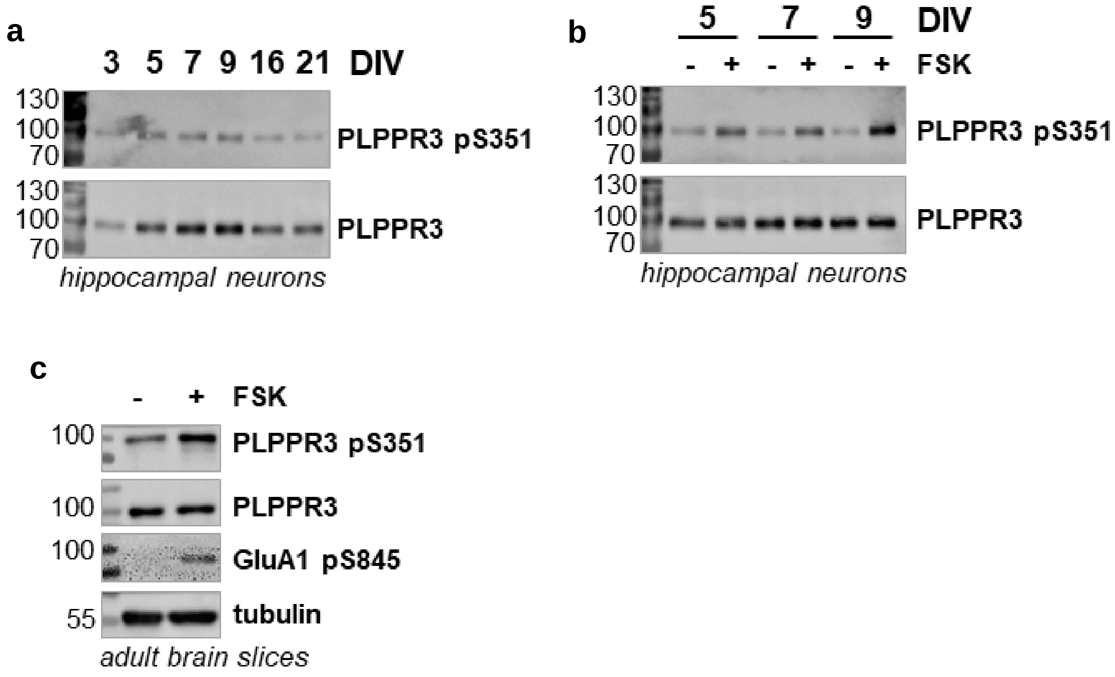

Figure S3. PLPPR3 is phosphorylated at S351 in neurons. Related to Figure 4. (a) PLPPR3 pS351 levels mimic total PLPPR3 levels through developmental stages. Primary hippocampal neurons were lysed at indicated timepoints and pS351 levels were analyzed using custom made antibody. (b) Phosphorylation of PLPPR3 S351 can be triggered in neurons. Primary cultured of (DIV5, 7 or 9) hippocampal neurons were stimulated with Forskolin (30 µM, 5 minutes), and analyzed by western blot. (c) Phosphorylation of PLPPR3 S351 can be triggered in adult brain tissue. Acute brain slices were stimulated with Forskolin (30 µM, 15 minutes), lysed and analyzed by western blot.

a

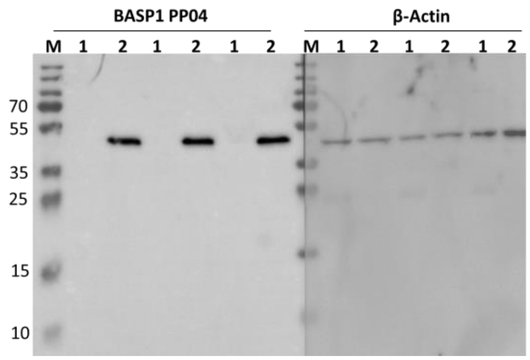

1. Kidney Lysate (Basp1- tissue)
2. Brain Lysate (Basp1+ tissue)

b

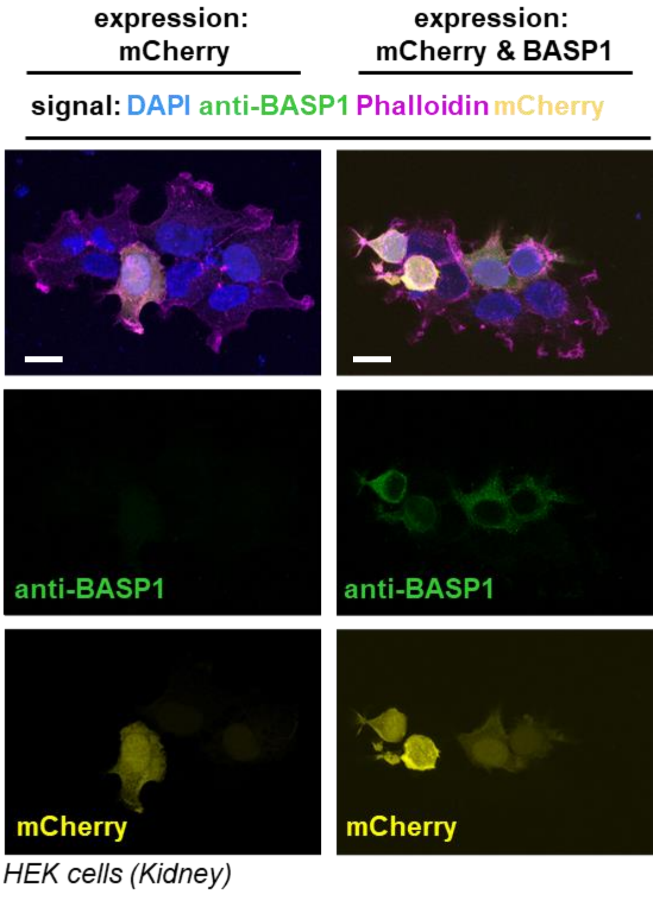

Figure S4. Validation of the custom made BASP1 antibody. Related to Figure 4. (a) Specificity of the BASP1 antibody was tested by western blot of protein lysates isolated from mouse kidney tissue (BASP1-negative) and mouse brain tissue (BASP1-positive). All antibody fractions were used at 1:500 dilution of the antibody. Note that the detected band of 50 kD is much higher than the predicted molecular weight of BASP1, which has been reported before.[60] (b) Specificity was tested by immunofluorescence of HEK293 cells transfected with control mCherry (left) and mCherry plus BASP1 expressing plasmids (right). Scale bars = 10 µm

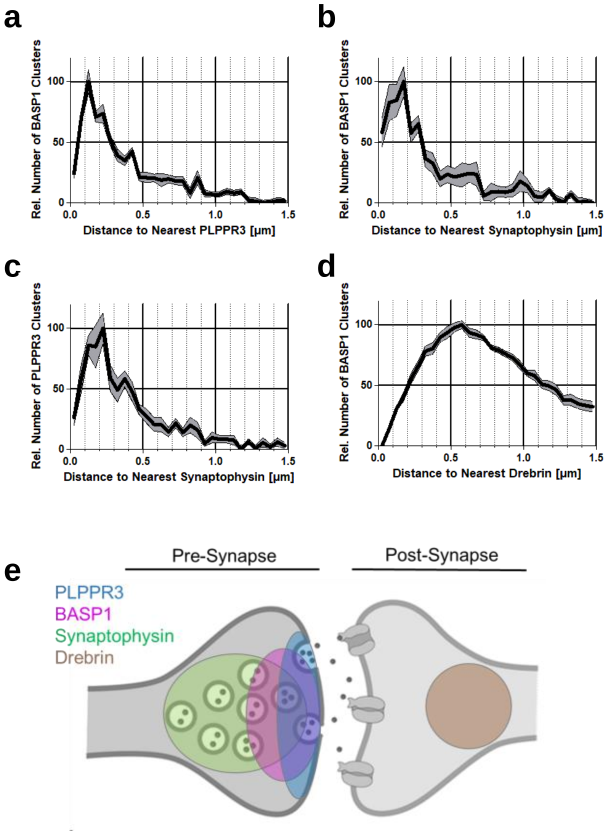

Figure S5. Nearest Neighbor (NN) analysis of BASP1 and PLPPR3 clusters with pre- and post-synaptic markers. Related to Figure 4. PLPPR3 and BASP1 co-cluster, as indicated by the NN peak of ~100 nm (a). Both proteins primarily localize near the presynaptic marker Synaptophysin (b, c; NN peaks ~200 nm). This presynaptic enrichment is further supported by the large separation between BASP1 and the post-synaptic marker Drebrin (d, NN peak ~600 nm). Mean ± SEM, N = 11 (a), 10 (b), 6 (c), 33 (d) representative images. (e) Schematic demonstrating distribution of markers according to known localization to pre- or postsynaptic compartments and obtained measurements.

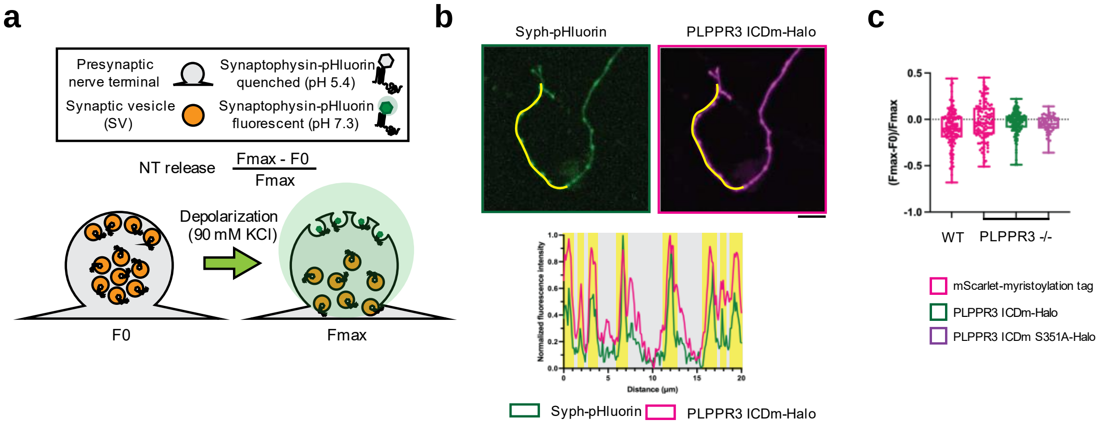

Figure S6. Live-cell imaging of primary hippocampal neurons expressing membrane-targeted PLPPR3 ICDm-Halo. Related to Figure 5. (a) Schematic representation of pHluorin-based assay used in this study. Primary hippocampus neurons are transfected with synaptophysin-pHluorin construct targeted to synaptic vesicles. Upon depolarization and synaptic vesicle fusion, the pH-sensitive luminal domain of synaptophysin-pHluorin is dequenched, which leads to the increase of fluorescence. (b) Top: Representative image of a stimulated wild-type neuron (DIV 14), expressing PLPPR3 ICDm-Halo and synaptophysin-pHluorin (Syph-pHluorin). Bottom: the line profile along the axon (yellow line in the image) indicating the fluorescence colocalization. Scale bar: 5 µm. (c) Quantification of fluorescence change upon KCl stimulation for constructs co-expressed with synaptophysin-pHluorin in assays used in this study (mScarlet-myristoylation tag, PLPPR3 ICDm-Halo and PLPPR3 ICDm S351A-Halo). Each datapoint represents a single analysed synaptic bouton. For each condition, data are from three independent biological replicates (N=3 independent neuronal cultures). Data are represented as mean ± SEM.
